# A Manganese Porphyrin Platform for the Design and Synthesis of Molecular and Targeted MRI Contrast Agents

**DOI:** 10.3390/ijms24119532

**Published:** 2023-05-31

**Authors:** Kyle D. W. Vollett, Daniel A. Szulc, Hai-Ling Margaret Cheng

**Affiliations:** 1 Institute of Biomedical Engineering, University of Toronto, Toronto, ON M5S 3G9, Canada; k.vollett@utoronto.ca (K.D.W.V.); Daniel.szulc@mail.utoronto.ca (D.A.S.); 2 Translational Biology & Engineering Program, Ted Rogers Centre for Heart Research, Toronto, ON M5G 1M1, Canada; 3 The Edward S. Rogers Sr. Department of Electrical & Computer Engineering, University of Toronto, Toronto, ON M5S 3G8, Canada

**Keywords:** magnetic resonance imaging (MRI), manganese porphyrin, molecular imaging, targeted contrast agent, albumin, collagen

## Abstract

Magnetic resonance imaging (MRI) contrast agents, in contrast to the plethora of fluorescent agents available to target disease biomarkers or exogenous implants, have remained predominantly non-specific. That is, they do not preferentially accumulate in specific locations in vivo because doing so necessitates longer contrast retention, which is contraindicated for current gadolinium (Gd) agents. This double-edge sword implies that Gd agents can offer either rapid elimination (but lack specificity) or targeted accumulation (but with toxicity risks). For this reason, MRI contrast agent innovation has been severely constrained. Gd-free alternatives based on manganese (Mn) chelates have been largely ineffective, as they are inherently unstable. In this study, we present a Mn(III) porphyrin (MnP) platform for bioconjugation, offering the highest stability and chemical versatility compared to any other T_1_ contrast agent. We exploit the inherent metal stability conferred by porphyrins and the absence of pendant bases (found in Gd or Mn chelates) that limit versatile functionalization. As proof-of-principle, we demonstrate labeling of human serum albumin, a model protein, and collagen hydrogels for applications in in-vivo targeted imaging and material tracking, respectively. In-vitro and in-vivo results confirm unprecedented metal stability, ease of functionalization, and high T_1_ relaxivity. This new platform opens the door to ex-vivo validation by fluorescent imaging and multipurpose molecular imaging in vivo.

## 1. Introduction

Contrast agents are chemical species that boost contrast-to-noise and improve the sensitivity of detection across a broad range of medical imaging modalities. From highlighting tumors to identifying ischemic or inflammatory tissue, contrast agents are an indispensable facet in modern radiology. Different modalities utilize different mechanisms for contrast generation. In magnetic resonance imaging (MRI), gadolinium (Gd), a paramagnetic metal, is used to augment signal contrast [1]. With seven unpaired electrons, Gd induces very efficient relaxation of longitudinal relaxation, but it is toxic in free, ionic form and must be chelated for sequestration and safe elimination from the body. To minimize risk of demetalation in vivo, all clinically approved Gd agents are designed for rapid elimination, with a half-life under 1.5 to 2 h in healthy subjects [2]. Longer-circulating Gd agents have also been used clinically, with a recent example being Ablavar, a blood-pool agent with an affinity for albumin and an elimination half-life of 18.5 h [3]. However, in 2017, Ablavar was unexpectedly withdrawn from the market by Lantheus Medical, citing poor sales; interestingly, the parent compound of Ablavar, Gd-DTPA, was internationally restricted even earlier due to risk of nephrogenic systemic fibrosis (NSF) [4]. Currently, there is no MRI blood-pool contrast agent manufactured for clinical imaging.

Unlike the vast majority of clinical Gd agents that are non-specific, Ablavar is an example of a targeted agent: that is, it has affinity for specific biological targets, in this case, albumin. Other targeted agents abound preclinically, all of them utilizing a bifunctional chelating agent (BFCA) to chelate Gd on the one hand and, on the other, to conjugate peptides, antibodies, or small organic molecules with affinity for specific targets [5,6]. However, none has been translated to the clinic. One possible explanation may be the prolonged retention required of any targeted contrast agent to accumulate at a specific tissue location, a requirement at odds with the need for rapid Gd elimination. A second explanation is that the stability of Gd complexes is not guaranteed when the elimination half-life exceeds 1.5 h. This concern around stability stems from a universal feature of Gd complexes, including the most inert macrocyclic ones: the pendant coordinating bases essential to metal chelation are susceptible to protonation by acid or endogenous biomolecules, ultimately releasing toxic free metal [7]. The much higher risk of toxicity posed by targeted contrast agents not designed to be eliminated quickly is the most plausible reason for the absence of Gd BFCA agents in the clinic.

New generations of Gd-free contrast agents have been investigated to address the limitations discussed above. Most commonly, manganese (II) (Mn^2+^), a trace dietary mineral with five unpaired electrons, has been explored given its paramagnetism. These Mn complexes, however, are labile and significantly underperform relative to Gd macrocyclic agents with respect to chemical inertness, as they rapidly demetalate in vivo [8,9]. Similar to their Gd counterparts, Mn(II) macrocyclic complexes also rely on pendant coordinating bases for metal chelation, thus suffering the same limitation of demetalation. While toxicity may not be as serious an issue, rapid signal loss from Mn release undermines the potential for targeted molecular imaging.

What is less appreciated is that these pendant coordinating bases limit chemical versatility, precluding the use of a vast array of bioconjugate methods available to fluorophores. The likelihood of metal release increases in acidic conditions or even in the presence of conjugative proteins, as both can interfere with pendant base coordination [10,11]. Furthermore, as pendant bases are usually nucleophilic, they are vulnerable to side reactions that compromise chelate stability. For this reason, BFCA synthesis [12] and conjugation are laborious processes, necessitating protection of pendant groups and restricting metal insertion to the final step [13,14]. This solution is far from ideal—metalation in the presence of large macromolecules leads to unstable metal complexation, and metalation can either be achieved for a fraction of conjugated complexes or not at all (e.g., solids and hydrogels).

Manganese (III) (Mn^3+^) complexes have much greater stability than Mn^2+^. However, with fewer electrons and lower valency, they are also generally associated with inadequate T_1_ relaxivity. Mn^3+^ porphyrins are exceptional in this regard, achieving higher T_1_ relaxivity at clinical field strengths than most current Gd or Mn(II) agents [15]. Yet, despite a long history of investigation as a Gd-free contrast agent, the potential of Mn(III) porphyrins as an improved BFCA platform for bioconjugation has not been fully explored. A possible reason may be the difficulty of metalation, which requires strong bases and high temperatures, both of which can degrade conjugated products [16] and limit their use in traditional pre-metalated conjugative approaches common to BFCAs. However, if sufficient stability could be achieved, pre-metalated approaches may be adopted to improve conjugation versatility. The potential of this approach is supported by a handful of previous research, where Mn(III) porphyrin amines were used as nucleophiles in EDC couplings [17] and Schiff base reactions [18,19].

In this work, we demonstrate that porphyrins provide a far superior Gd-free platform for MRI bioconjugation. Specifically, we developed an electrophilic Mn porphyrin platform that affords conjugation versatility, high stability, very low risk of toxicity, and facile one-step conjugation to proteins. For the first time, we prove that sodium Mn(III) 5-(4-aminophenyl)-10,15,20-(tri-4-sulfonatophenyl)porphyrin chloride (MnTPPS_3_NH_2_) exceeds the kinetic stability of any known Gd- or Mn-based contrast agent. MnTPPS_3_NH_2_ can then be functionalized with isothiocyanate, an electrophilic moiety ubiquitous in fluorescent bioconjugation, to form sodium Mn(III) 5-(4-phenylisothiocyanate)-10,15,20-(tri-4-sulfonatophenyl)porphyrin chloride (MnTPPS_3_NCS), for which we report the first successful synthesis and characterization. To demonstrate the conjugation versatility of our new Mn BFCA—MnTPPS_3_NCS, we use it to covalently label human serum albumin (HSA), a model protein, and collagen hydrogel, a common scaffolding material in tissue engineering applications.

## 2. Results and Discussion

### 2.1. Stability of MnTPPS_3_NH_2_

Low toxicity of the MnTPPS_3_NH_2_ precursor has previously been demonstrated on different cell lines [20,21], including human embryonic stem cells [22]. More generally, sulfonated porphyrins have been shown to remain intact in vivo and after elimination from the body in many animal studies [23,24]. The high thermodynamic stability of Mn^3+^ porphyrins is well established, with high kinetic stability of MnTPPS_3_NH_2_ specifically suggested in our previous experience in material labeling [18,20,25]. In this study, we aimed to confirm its kinetic stability before attempting to convert the single aromatic amine on a pre-metalated porphyrin to an isothiocyanate electrophile. In the following, we provide evidence in support of high stability: kinetic inertness was maintained at low pH, and trans-metalation (leading to Mn^2+^ dissociation) was negligible even over long intervals in the presence of competitive metals, even at low pH.

**Figure 1:**
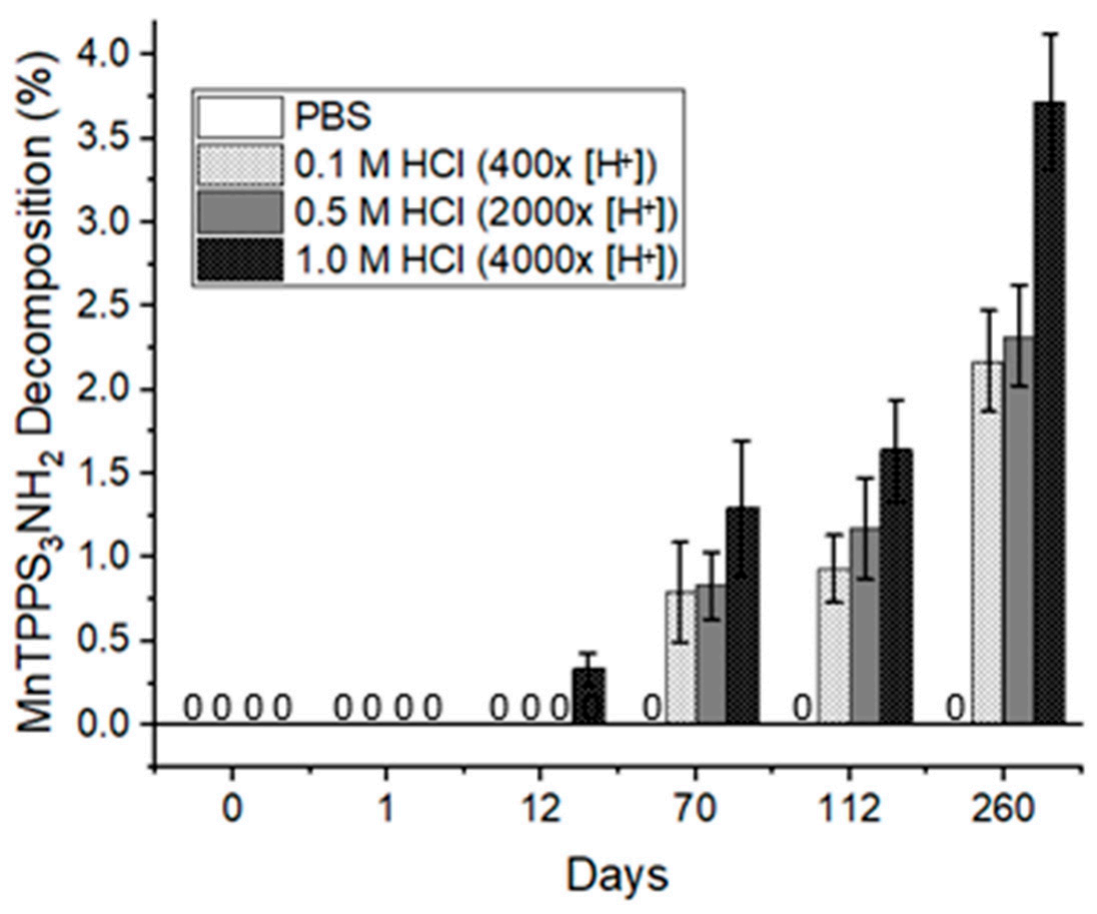
**High kinetic inertness of MnTPPS_3_NH_2_ in acidic solutions over 260 days.** Percentage demetalation of MnTPPS_3_NH_2_ measured by the relative concentration of metal-free TPPS_3_NH_2_ detected by UHPLC (*n* = 2).

To create the MRI bioconjugation platform, commercially available porphyrin TPPS_3_NH_2_ was metalated with slight modification of an established procedure [18], which is outlined in Supplementary Scheme S1. The structure and purity of the resulting MnTPPS_3_NH_2_ were determined by ultraviolet–visible (UV–VIS) spectra, high performance liquid chromatography (HPLC), flame atomic absorption spectroscopy (FAAS), and mass spectrometry (ESI-MS) (Supplementary Figures S1–S3). The kinetic inertness of MnTPPS_3_NH_2_ was confirmed using an acid decomposition test (Figure 1), a method used to predict the stability of Gd contrast agents in vivo [26]. In control PBS solution, demetalation was absent over the 260-day study, a finding that places the stability of MnTPPS_3_NH_2_ much higher than that of most Mn^2+^ chelates, which decompose in PBS. No Mn^3+^ release by MnTPPS_3_NH_2_ was detectable when stored in PBS or HSA (0.6 mM, PBS) after 260 days. Stress testing in 0.1 (400× competitive [H^+^] excess) and 0.5 M HCl (2000× competitive [H^+^] excess) (note: measurements are typically performed at pH = 1, or 0.1 M HCl) [26] resulted in minimal Mn^3+^ release: less than 1% after 112 days. Only at a 10-fold increase of HCl (at 1 M) was trace demetalation detected earlier by day 12. Linearly extrapolating the rate of decay suggests that the dissociation half-life (T_1/2_) for MnTPPS_3_NH_2_ exceeds 17, 15, and 10 years at HCl concentrations of 0.1, 0.5, and 1 M, respectively. By comparison, we found that Gadovist, the most common clinical MRI contrast agent, completely dissociated by 48 h when subjected to 0.1 M HCl at 37 °C (Figure S4). This is consistent with most stable clinical Gd MRI agents, whose half-lives range between 7 to 24 h under equivalent conditions [27,28]. Faring even more poorly, a majority of Mn^2+^ chelates cannot match the kinetic stability of many linear Gd chelates, with acid decomposition half-lives in the range of seconds [29,30]. Even the most kinetically stable Mn^2+^ chelate for use as an MRI contrast agent demonstrated to date was found to have a decomposition half-life of 70 min when subjected to 0.1 M HCl (100× competitive [H^+^] excess) at 25 °C [31].

**Figure 2:**
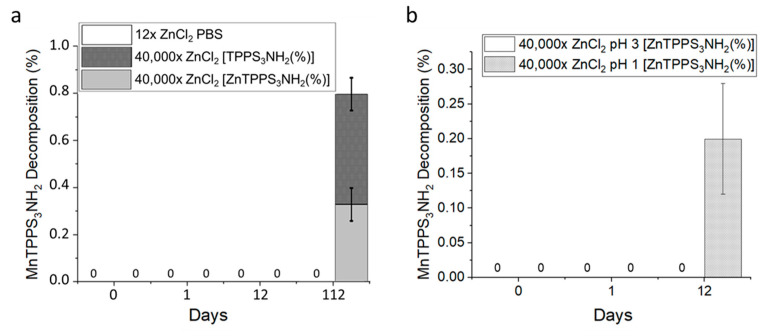
**High kinetic inertness of MnTPPS_3_NH_2_ against trans-metalation by Zn^2+^ in neutral and acidic conditions.** Percentage of MnTPPS_3_NH_2_ decomposition represents the total amount of demetalation or trans-metalation of MnTPPS_3_NH_2_ measured by relative concentration of either TPPS_3_NH_2_ or ZnTPPS_3_NH_2_, respectively, by UHPLC (*n* = 2). (**a**) Resistance of MnTPPS_3_NH_2_ against trans-metalation and demetalation during ZnCl_2_ stress tests over 112 days. (**b**) Resistance of MnTPPS_3_NH_2_ against trans-metalation and demetalation during combined ZnCl_2_ and acid stress tests.

Trans-metalation with endogenous metal ions (e.g., Zn^2+^, Cu^2+^, and Fe^3+^) is another means by which the chelated metal is exchanged and released. Most attention is directed towards Zn^2+^ due to its higher relative abundance in plasma (50 µM). Therefore, ZnCl_2_ stress testing is critical for assessing kinetic stability under physiological conditions. Figure 2a confirms the high kinetic inertness of MnTPPS_3_NH_2_ against trans-metalation. Incubation of MnTPPS_3_NH_2_ in a 12× excess of ZnCl_2_ (i.e., 3 mM ZnCl_2_ in PBS) did not result in detectable decomposition even after 112 days. At a 40,000× excess of ZnCl_2_ (10 M in DI water, pH 5.5), only 0.33% trans-metalation and 0.47% demetalation were detected by 112 days. A high resistance against trans-metalation was expected given that both trans-metalation and demetalation of porphyrins were previously reported to be primarily mediated by acid hydrolysis [13]. MnTPPS_3_NH_2_ was demonstrated to maintain impressive resistance to decomposition in conditions of combined acid and ZnCl_2_ stress in Figure 2b, where only 0.2% trans-metalation was detected at pH 1 after 12 days and none at a pH of 3. This is notable given that even the most trans-metalation-resistant Mn^2+^ chelates lose this resistance in acid and rapidly degrade as the pH approaches 3 and their pendant metal coordinating bases become protonated [31].

The results in Figure 1 and Figure 2 demonstrate the unmatched inertness of the MnTPPS_3_NH_2_ complex to acid decomposition, ZnCl_2_ stress test, and the combination of both, surpassing all current Mn^2+^ and Gd-based chelates. A survey of the Gd contrast agent literature reveals that Gd-DOTA and other macrocyclic chelates are considered stable against trans-metalation by Zn^2+^ at physiological conditions when their pendant coordinating bases are unprotonated, but these studies were conducted within one week [32,33]. One study of Gd-DOTA reported a half-life of 100 h in the presence of 100× Zn^2+^ [34], which suggests the agent would not stand up to the long interval of testing done in our study. These stability results support that MnTPPS_3_NH_2_ may be ideal for applications requiring extended residence times in vivo. Furthermore, they support the notion that MnTPPS_3_NH_2_ can be functionalized with isothiocyanate without risk of demetalation.

### 2.2. Electrophilic Functionalization of MnTPPS_3_NH_2_

**Scheme 1:**
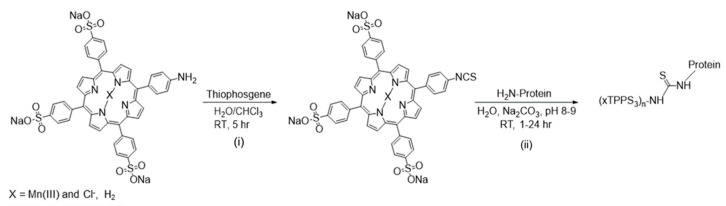
Functionalization of MnTPPS_3_NH_2_ to the electrophilic MnTPPS_3_NCS conjugation platform (**i**), followed by generalized protein conjugation by the isothiocyanate-functionalized Mn porphyrin platform (**ii**).

Scheme 1i illustrates our conversion of the amine on MnTPPS_3_NH_2_ or TPPS_3_NH_2_ to an isothiocyanate. This led to a near quantitative conversion of isothiocyanate-functionalized MnTPPS_3_NCS at high purity according to UV–VIS spectra, HPLC, FAAS, and ESI-MS and Fourier transform infrared spectroscopy (FTIR-ATR) (Supplementary Figures S5–S9a). The Mn-free TPPS_3_NCS was also synthesized and characterized as above with the addition of a ^1^HNMR spectrum (Supplementary Figures S9b–S13).

It is worth noting that the advantages of a pre-metalated conjugation approach were previously recognized. An isothiocyanate-functionalized Gd (1,4,7,10-tetraazacyclododecane-*N*,*N*′,*N*″,*N*‴-tetraacetic acid) derivative (Gd-DOTA-NCS) was synthesized to achieve bioconjugation with proteins and macromolecules [10,11,35]. This functionalization was only possible because Gd-DOTA-based derivatives are the most inert commercial Gd chelates and are sufficiently stable under acidic conditions during their brief conversion to an isothiocyanate. In seeking a Gd-free alternative, other metal chelates, particularly Mn^2+^, are insufficiently stable in acid to undergo isothiocyanate functionalization. Other methods of electrophilic activation that do not require acidic conditions, such as carbodiimide and succinimides, are also not viable, because their pendant nucleophiles necessary for metal chelation are unprotected [36,37,38]. Without the use of a protective group, pendant bases are vulnerable to activation and conjugation in carbodiimide and succinimide chemistry. This leads to side reactions where these bases reduce the coordination number and stability of a fraction of chelates. The consequence is that purified conjugated macromolecules may contain different chelate species resulting from side reactions.

### 2.3. Conjugation to Human Serum Albumin (HSA) and MR Imaging

As proof-of-principle, HSA, a protein ubiquitously demonstrated in most bioconjugate techniques, was used to exemplify bioconjugation of MnTPPS_3_NCS to proteins. Scheme 1ii depicts the general scheme; conditions adopted were common for conjugating isothiocyanates to proteins. It is possible to conjugate more than one MnTPPS_3_NCS to a single HSA. This would be beneficial, for example, if one wishes to achieve a higher longitudinal (T_1_) relaxivity for maximum detection sensitivity. Coupling efficiency was first ascertained at various stoichiometries, with reactions performed with 5×, 10×, 20×, and 40× excess of MnTPPS_3_NCS to HSA. To rule out the possibility of non-specific binding of porphyrin to HSA, concurrent reactions at all relevant equivalencies were performed between MnTPPS_4_ and HSA. The resulting degree of labeling and tagging efficiencies for both MnTPPS_3_NCS and control MnTPPS_4_ are shown in Table 1.

MnTPPS_3_NCS conjugation to HSA produced a notably high tagging efficiency of 60-70% for the lowest tagging equivalences tested (5× and 10× excess of Mn), leading to 3.5 and 6 MnTPPS_3_ per HSA, respectively. This tagging efficiency decreased to 48% at higher excesses, yielding 9.5 and 19 MnTPPS_3_ per HSA. Non-specific binding from MnTPPS_4_ resulted in negligible tagging. Our results can be compared to fluorescent and chelating platforms for HSA conjugation (see Tables S1 and S2 in the Supplementary materials). The efficiency of MnTPPS_3_NCS for achieving high protein loading is particularly important in applications where the biological target concentration falls below the limit of detection on MRI for small molecule T_1_ contrast agents.

**Figure 3:**
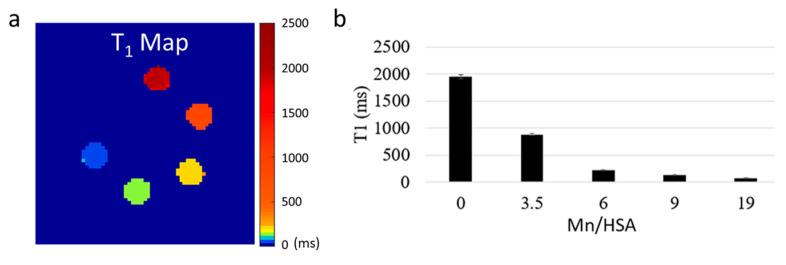
**T_1_ relaxation time measurements of HSA-conjugated MnP contrast agents.** (**a**) T_1_ map of doped solutions: HSA control (top) followed clockwise by (MnTPPS_3_)_3.5_HSA, (MnTPPS_3_)_6_HSA, (MnTPPS_3_)_9_HSA, and (MnTPPS_3_)_19_HSA. (**b**) T_1_ measurements for HSA control (0), (MnTPPS_3_)_3.5_HSA, (MnTPPS_3_)_6_HSA, (MnTPPS_3_)_9_HSA, and (MnTPPS_3_)_19_HSA. Shown are mean T_1_ values and standard deviation.

In-vitro MRI scans on a clinical 1.5-Tesla scanner confirmed a large reduction in T_1_ relaxation times induced by the HSA-conjugated contrast agents (Figure 3). Relaxation rate scaled linearly with the degree of tagging, producing a relaxivity of 15.4 mM^−1^s^−1^ when using 0.05 mM of bound MnTPPS_3_ (Supplementary Figure S14). These results suggest that MnTPPS_3_NCS is an exceptional platform for protein conjugation and can achieve a high degree of labeling to achieve high sensitivity.

**Figure 4:**
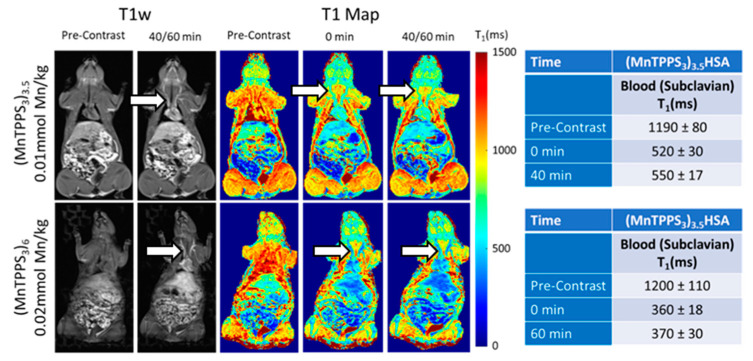
**MRI of healthy rats injected with blood-pool MnP contrast agents.** In-vivo T_1_-weighted (T1w) spin-echo images and T_1_ maps of longitudinal relaxation times in healthy rats injected with (MnTPPS_3_)_3.5_HSA or (MnTPPS_3_)_6_HSA. Respective tables of T_1_ and T_2_ relaxation times in blood (carotid (arrow) and subclavian arteries) are shown.

In-vivo MRI scans of rats on a clinical 1.5-Tesla scanner injected intravenously with the blood-pool agents demonstrate a large T_1_ reduction in the intravascular space (Figure 4). (MnTPPS_3_)_3.5_HSA and (MnTPPS_3_)_6_HSA were compared for their contrast efficiency and time-course signal change. Both agents provided stable T_1_ in blood for at least one hour. A greater T_1_ reduction was seen for (MnTPPS_3_)_6_HSA due to higher metal loading per HSA molecule.

### 2.4. Stability of (MnTPPS_3_)_6_HSA

**Figure 5:**
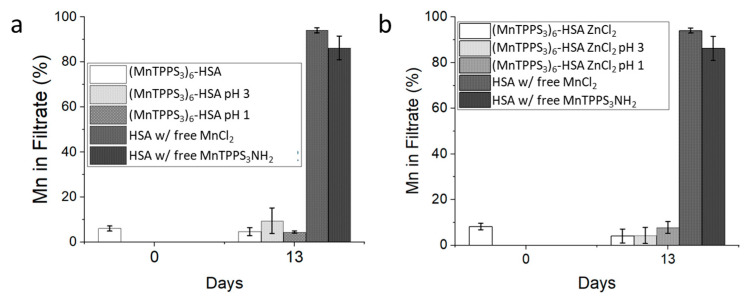
**High stability of (MnTPPS_3_)_6_-HSA under ambient, acid, and/or ZnCl_2_ stressed conditions.** Percentage of Mn removed from samples of (MnTPPS_3_)_6_-HSA by ultrafiltration represents the cumulative total of free Mn, MnTPPS_3_ decoupled from HSA, and (MnTPPS_3_)_6_-HSA that bypassed the filter with Mn measured on ICP-OES (*n* = 2). (**a**) Acid stress testing of (MnTPPS_3_)_6_-HSA for 13 days. (**b**) ZnCl_2_ and combined ZnCl_2_ and acid stress testing of (MnTPPS_3_)_6_-HSA for 13 days.

The stability of the protein-conjugated MnTPPS_3_NCS linkage was also tested. (MnTPPS_3_)_6_HSA was subjected to a series of acid and zinc stress tests at 37 °C: Distilled water, HCl solutions at pH = 1 and 3, and ZnCl_2_ solutions at 40,000× equivalence in either distilled water or HCl solutions (pH = 1 and 3). After 13 days, solutions were spin filtered to remove decoupled Mn porphyrin and free Mn. Figure 5 shows the cumulative total free and decoupled Mn porphyrin for various stress tests. No significant (MnTPPS_3_)_6_HSA decomposition was detected over the course of 13 days under any condition tested, which is consistent with our results for MnTPPS_3_NH_2_. This indicates that conjugates of MnTPPS_3_NCS remained inert to demetalation and trans-metalation. Furthermore, the thiourea linkage remained intact over 13 days in both physiological and highly acidic conditions.

### 2.5. Labeling of Collagen

Targeting need not be limited to albumin or other endogenous entities. In fact, targeting materials has immense value in tissue engineering and regenerative medicine, and examples of material labeling with fluorescence abound. In contrast, material labeling with T_1_ agents is rare, and existing reports do not employ a covalent mechanism to permit equating signal loss to material degradation. In the following, we demonstrate the first covalent labeling of collagen for material tracking on T_1_-weighted MRI.

**Figure 6:**
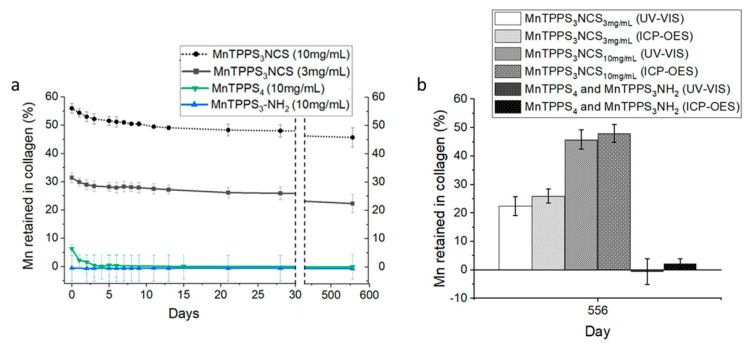
**Stability of MnTPPS_3_NCS-labeled collagen hydrogels over 556 days after labeling.** (**a**) Percentage of MnTPPS_3_ released from labeled collagen gels (*n* = 2), according to UV–VIS. The stabilized % Mn retained in collagen represents tagging efficiency. (**b**) Validation of UV–VIS results for MnTPPS_3_NCS-labeled collagen with ICP-OES performed on digested gels and solutions at 556 days.

Hydrogels composed of either 3 mg/mL or 10 mg/mL collagen were labeled in situ with MnTPPS_3_NCS. This procedure was repeated for both MnTPPS_4_ and MnTPPS_3_NH_2_ (two different controls) to account for possible retention of porphyrin due to non-specific binding. The retention of porphyrins in collagen was monitored over the course of 556 days (Figure 6). Labeling with MnTPPS_3_NCS yielded much higher retention of MnTPPS_3_ (56 ± 2% of total reacted MnTPPS_3_NCS) relative to controls MnTPPS_4_ and MnTPPS_3_NH_2_, where the porphyrins were mostly removed after washing at day 0 (Figure 6a). In fact, most MnTPPS_3_ release occurred over the initial 5 days (Supplementary Figure S15), which is likely attributable to the release of residual free porphyrin. To further validate the results of MnTPPS_3_NCS binding, collagen samples were digested in nitric acid and assessed for Mn content by inductively coupled plasma–optical emission spectroscopy (ICP-OES) alongside their respective incubation and wash solutions (Figure 6b). ICP-OES results demonstrated the stability of MnTPPS_3_NCS collagen conjugates, with 3 mg/mL gels retaining 22 ± 3% (UV–VIS) and 26 ± 2% (ICP-OES), and 10 mg/mL gels retaining 46 ± 3% (UV–VIS) and 48 ± 3% (ICP-OES). Controls had no porphyrin retained, and the combined retention of MnTPPS_4_ and MnTPPS_3_NH_2_ was only −0.6 ± 4% (UV–VIS) and 2 ± 1.6% (ICP-OES).

The above results clearly support the value of covalent binding in tracking material degradation. It is also worth mentioning that our pre-metalation approach is necessary in material labeling for a simple reason: metalation and removal of free metal, as required in conventional post-metalation, is impractical—even impossible—for viscous solutions and solids.

### 2.6. Labeling Acid-Soluble Collagen and MR Imaging

**Figure 7:**
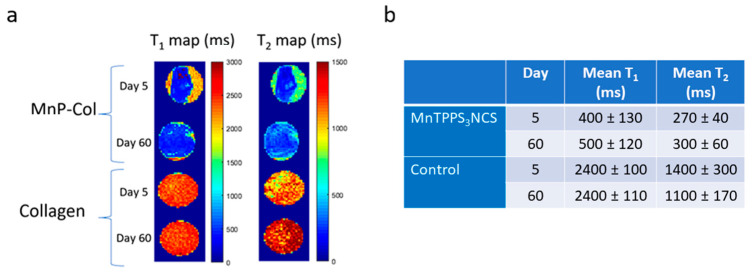
**T_1_ and T_2_ relaxation time measurements of collagen gels labeled with MnP-Col.** (**a**) T_1_ and T_2_ maps of collagen gels labeled with MnP-Col and collagen controls (i.e., unlabeled) at 5 and 60 days post-labeling. (**b**) T_1_ and T_2_ measurements represented as mean values and standard deviation.

Pre-labeled, acid-soluble collagen offers more versatility for in-situ labeling, expanding the range of applications to include cell-compatible gelation and injectable hydrogels. To further demonstrate the versatility MnTPPS_3_NCS, we synthesized MnTPPS_3_ tagged acid-soluble collagen, adopting methods used in collagen labeling with fluorescence [39]. This T_1_ reducing acid-soluble collagen can be stored and used later, similar to acid-soluble collagen, and can be blended with regular collagen. The method of labeling is akin to the in-situ conjugation procedure above, except that immediately after labeling of hydrogels, the labeled collagen is redissolved in 0.5 M acetic acid and dialyzed for 1 week to remove excess salt and free MnTPPS_3_ (Figure S39). Notably, this dialysis process requires extensive time at pH < 3, which would likely lead to significant demetalation in most other T_1_ contrast agents. However, our stability results for both MnTPPS_3_NH_2_ and (MnTPPS_3_)_6_HSA in acid suggest that collagen-conjugated MnTPPS_3_ is highly unlikely to demetalate in one week. To test the T_1_ efficiency of MnTPPS_3_NCS-tagged, acid-soluble collagen, purified dry MnTPPS_3_NCS-tagged, acid-soluble collagen was blended with unlabeled collagen at a 25% (*v*/*v*) to produce T_1_-labeled hydrogels 5 and 60 days before imaging on a clinical 3-Tesla scanner. T_1_- and T_2_-weighted images and quantitative T_1_ and T_2_ relaxometry maps are shown in Figure 7. A significant reduction of T_1_ and T_2_ relaxation times could be detected for MnTPPS_3_NCS-labeled collagen hydrogels relative to unlabeled collagen, with both T_1_ and T_2_ remaining low after 60 days.

## 3. Materials and Methods

Materials: *N*,*N*-diisopropylethylamine (DIPEA), manganese chloride (MnCl_2_), dimethylformamide (DMF), acetonitrile (ACN), phosphate-buffered saline (PBS), ammonium acetate (NH_4_OAc), lyophilized albumin from human serum powder (99%), sodium bicarbonate (NaHCO_3_), sodium carbonate (Na_2_CO_3_), nitric acid 70% (HNO_3_), and manganese standard for ICP were purchased from Sigma Aldrich (Steinheim, Germany). Pre-treated regenerated cellulose dialysis tubing (MWCO: 1 kD) and (MWCO: 50 kD) was purchased from Spectrum Labs (Cincinnati, OH, USA). Ion-exchange resin (amberlite IR120, H form) was purchased from ACROS Organics (Geel, Belgium). C-18 silica gel spherical (0.7–0.9 cm^3^/g pore volume, Supelco) was purchased from Sigma Aldrich. 5-(4-Aminophenyl)-10,15,20-(triphenyl)porphyrin was purchased from PorphyChem (Dijon, France). All chemicals were of proper analytical grade and were used without further purification.

### 3.1. Synthesis of Isothiocyanate-Functionalized Manganese Porphyrin (MnTPPS_3_NCS)

The metalated mono-amine porphyrin (MnP-NH_2_) (300 mg, 0.3 mmol) was dissolved in ultrapure water (37 mL) in a round bottom flask. Next, a solution of thiophosgene (276 µL, 3.6 mmol) dissolved in 3.5 mL chloroform was slowly added dropwise under vigorous stirring. The reaction remained at room temperature (RT) for 5 h until complete. The reaction was monitored by thin layer chromatography (TLC Silica Gel 60 F254, Millipore, Darmstadt, Germany) using a chloroform:methanol:water 6:3:1 mobile phase with 1% *v*/*v* of 1 N hydrochloric acid. Rf for MnTPPS_3_NCS was 0.68 while the MnTPPS_3_NH_2_ starting material was 0.52. The reaction solution was then washed six times with chloroform (100 mL) to remove remaining thiophosgene. The aqueous phase containing sodium manganese(III) 5-(4-isocyanatophenyl)-10,15,20-(tri-4-sulfonatophenyl)porphyrin chloride was then dried by lyophilization to form a green powder and stored for future use. Yield by weight was 99%. MS (ESI) *m*/*z* calculated for [M_2_-6H]^4–^ ((C_45_H_24_N_5_O_9_S_4_Mn)_2_)^4−^: 480.4924. Found: 480.4918 and is consistent with dimers formed by sulfonatophenyl metalloporphyrins [40] ε = 89196 M^−1^cm^−1^ max at 467 nm consistent with a Soret band associated with Mn(III) porphyrins [41].

### 3.2. Synthesis of Isothiocyanate-Functionalized Unmetalated Porphyrin (TPPS_3_NCS)

The unmetalated 5-(4-aminophenyl)-10,15,20-(tri-4-sulfonatophenyl)porphyrin (APO-NH_2_) trisodium salt was used to synthesize 5-(4-isocyanatophenyl)-10,15,20-(tri-4-sulfonatophenyl)porphyrin (APO-NCS) using an identical procedure as stated above. Rf for TPPS_3_NCS was 0.76 while TPPS_3_NH_2_ starting material was 0.66. Yield by weight (99%) ^1^H NMR (500 MHz, DMSO-*d*_6_) δ (ppm) 8.86 (m, 8H, β-pyrrole), 8.30 (m, *J* = 7.9 Hz, 2H), 8.19 (m, 6H), 8.06 (m 6H), 7.89 (d, 2H, *J* = 7.9 Hz), −2.94 (s, 2H). MS (ESI) *m*/*z* calculated for [M-3H]^2–^ C_45_H_27_N_5_O_9_S_4_^−2^: 454.53. Found: 454.53. ε = 224272 M^−1^cm^−1^ max at 419 nm.

### 3.3. Characterization of TPPS_3_NH_2_, MnTPPS_3_NH_2_, ZnTPPS_3_NH_2_, MnTPPS_4_, TPPS_3_NCS, MnTPPS_3_NCS

The identity and purities of TPPS_3_NH_2_, MnTPPS_3_NH_2_, ZnTPPS_3_NH_2_, MnTPPS_4_, TPPS_3_NCS, and MnTPPS_3_NCS were determined by UV–VIS spectroscopy, ^1^H NMR, HPLC, ICP-OES, and mass spectroscopy. UV–VIS spectra were recorded on a Beckman Coulter DU 800 UV–VIS spectrophotometer (Brea, CA, USA). Absorption spectra are provided in supplementary Figures S1, S5, S10, and S16–S18 and were measured in PBS buffer at 25 °C for MnTPPS_3_NH_2_ (λ_max_ = 467 nm, ε = 74,529 M^−1^cm^−1^), MnTPPS_3_NCS (λ_max_ = 466 nm, ε = 89,196 M^−1^cm^−1^), TPPS_3_NCS (λ_max_ = 413 nm, ε = 224,272 M^−1^cm^−1^), TPPS_3_NH_2_ (λ_max_ = 414 nm, ε = 231,885 M^−1^cm^−1^), ZnTPPS_3_NH_2_ (λ_max_ = 420 nm, ε = 243,300 M^−1^cm^−1^), and MnTPPS_4_ (λ_max_ = 464 nm, ε = 80,523 M^−1^cm^−1^). HPLC spectra for TPPS_3_NH_2_, MnTPPS_3_NH_2_, TPPS_3_NCS, and MnTPPS_3_NCS were recorded using a Perkin Elmer Series 200 system (Waltham, MA, USA) with UV/Vis detector recording at both 419 and 467 nm using a gradient of 10 mM ammonium acetate buffer (NH_4_OAc) and acetonitrile. A Supelco Supercosil LC-18 column with dimensions 25 cm × 4.6 mm and 5 µm beads was used at the University of Toronto Department of Chemistry ANALEST facility. MnTPPS_3_NH_2_ and TPPS_3_NH_2_ eluted at 8.41 min with 93.37% purity and 9.45 min with 93.39% purity, respectively (Supplementary Figures S2 and S19). MnTPPS_3_NCS and TPPS_3_NCS eluted at 13.94 min with 91.8% purity and 11.39 min with 94.25% purity, respectively (Supplementary Figures S6 and S11). Concentrations and confirmation that free Mn and Zn were removed was determined by Thermo Scientific iCAP Pro ICP-OES (Madison, WI, USA), measuring absorption at 257.610 nm and 213.856 nm at the University of Toronto Department of Chemistry ANALEST facility with MnTPPS_3_NH_2_ 87.02%, MnTPPS_3_NCS 88.11%, and ZnTPPS_3_NH_2_ 86.79%. Mass spectroscopy was performed with an Agilent 6538 Q-TOF (Santa Clara, CA, USA) in ESI MS Negative or Positive modes at the University of Toronto Department of Chemistry AIMS Mass Spectrometry Laboratory (Supplementary Figures S3, S7, S12 and S20). MS (ESI) *m*/*z* calculated for [M_2_-6H]^4–^ ((C_44_H_26_N_5_O_9_S_3_Mn)_2_)^4−^: 459.5142. Found: 459.5137 for MnTPPS_3_NH_2_. MS (ESI) *m*/*z* calculated for [M]^+^ C_45_H_27_N_5_O_9_S_4_Mn^+^: 964.01. Found: 964.00 for MnTPPS_3_NCS. MS (ESI) *m*/*z* calculated for [M_2_-6H]^4–^ ((C_45_H_24_N_5_O_9_S_4_Mn)_2_)^4−^: 480.4924. Found: 480.4918 for MnTPPS_3_NCS. MS (ESI) *m*/*z* calculated for [M-2H]^2–^ C_45_H_27_N_5_O_9_S_4_^−2^: 454.5351. Found: 454.5347 TPPS_3_NCS. MS (ESI) *m*/*z* calculated for [M_2_-6H]^2–^ ((C_44_H_27_N_5_O_9_S_3_Zn)_2_)^2−^: 464.5137. Found: 464.5135 for ZnTPPS_3_NH_2_ Fourier transform infrared spectroscopy (FTIR-ATR, Thermo Scientific Nicolet iS50, Madison, WI, USA) was performed on MnTPPS_3_NH_2_, MnTPPS_3_NCS, TPPS_3_NH_2_, and TPPS_3_NCS with spectra included in Supplementary Figure S9 with isothiocyanate products having an absorption peak at 2050 cm^−1^ associated with isothiocyanate stretching. ^1^H NMR spectra of TPPS_3_NCS and TPPS_3_NH_2_ were recorded on a Bruker US 500 MHz system (Santa Clara, CA, USA) (Figures S13 and S21) at the University of Toronto Department of Chemistry CSICOMP NMR facility.

### 3.4. Stability of Acid-Stressed MnTPPS_3_NH_2_ against De-Metalation

The stability of MnTPPS_3_NH_2_ against demetalation was tested in neutral and increasingly acidic solutions. Stock solution of MnTPPS_3_NH_2_ was mixed with HCl or water in two replicates each. Resulting solutions were composed of 0.25 mM MnTPPS_3_NH_2_ with either PBS, PBS and 0.6 mM HSA, 0.1 M HCl, 0.5 M HCl, or 1.0 M HCl. Solutions were stored in an incubator at 37 °C for either 1, 12, 70, 112, or 260 days. The sample condition of PBS and 0.6 mM HSA was only tested at *t* = 0 and after 260 days. These samples were then diluted in 10% acetonitrile and immediately assessed by the Waters ACQUITY H-class UHPLC system with PDA detector and single quadrupole MS detector at the University of Toronto Department of Chemistry ANALEST facility. This was compared to a series of standards measured for MnTPPS_3_NH_2_, TPPS_3_NH_2_, and ZnTPPS_3_NH_2_ (Supplementary Figures S22–S24). These three porphyrins were also mixed to ensure adequate separation (Supplementary Figure S25). Examples of UHPLC spectra for sample conditions are included in Supplementary Figures S26 and S27.

### 3.5. Stability of Zinc-Stressed MnTPPS_3_NH_2_ against Trans-Metalation

The stability of MnTPPS_3_NH_2_ against demetalation and trans-metalation by ZnCl_2_ was tested in neutral and increasingly acidic solutions. Concentrated stock solutions of MnTPPS_3_NH_2_ and ZnCl_2_ were first mixed in either distilled water or PBS, resulting in *n =* 2 solutions of 0.25 mM MnTPPS_3_NH_2_ with either PBS and 3 mM ZnCl_2_ (12×) or distilled water and 10 M ZnCl_2_ (40,000×). Acidic samples were similarly prepared from concentrated stock solutions before HCl was added, making samples comprising 10 M ZnCl_2_ (40,000×) with either 0.1 M or 0.003 M HCl in two replicates each. The resulting solutions were stored in an incubator at 37 °C, where zinc-stressed samples were tested at 1, 12, or 112 days. These samples were diluted in 10% acetonitrile immediately before being assessed by UHPLC and compared to standards. Examples of UHPLC spectra for sample conditions are included in Supplementary Figures S28 and S29.

### 3.6. Conjugation of MnTPPS_n_NCS to HSA

MnTPPS_3_NCS was added at different stoichiometric excess (0×, 5×, 10×, 20×, 40×, and 80×) to aqueous HSA solutions under vigorous stirring. Sodium carbonate buffer (0.1 M) solution was added to reaction until the pH reached 9 and was left for 24 h. Purification of (MnTPPS_3_)_n_-HSA was performed with 50 mL ultra spin filters (Amicon Ultra Regenerated cellulose 50 kDa MWCO; Millepore, Darmstadt, Germany) for 5 min at 4000 rpm to remove unbound porphyrin. Reaction solutions were repeatedly spin filtered with PBS well past the point that the resulting filtrate was transparent (9×). Following this, 3× more filtrations were completed with deionized water to remove PBS salt. Reaction solutions where then lyophilized producing a green colored solid that could be dissolved and used directly as intravascular contrast agent. Matrix-assisted laser desorption/ionization (MALDI) was used to determine MnTPPS_3_NCS tagging efficiency by change in average molecular weight of HSA. As a control to account for possible non-specific absorption of porphyrin; this entire synthesis procedure was also performed with MnTPPS_4_.

### 3.7. Characterization of MnTPPS_n_NCS and MnTPPS_4_ Labelled has

The degree of conjugation was determined by a Bruker Autoflex Speed matrix-assisted laser desorption ionization time-of-flight mass spectrometer (MALDI-TOF/MS) at the University of Toronto Department of Chemistry AIMS Mass Spectrometry Laboratory (Supplementary Figures S30 and S31). MnTPPS_4_ was used as a control for non-covalent absorption to HSA. Concentration and confirmation that free MnTPPS_3_ was removed was determined with a AAnalyst 100 system (PerkinElmer, Waltham, MA, USA) with a Manganese Lumina Hollow Cathode Lamp at the University of Toronto Department of Chemistry ANALEST facility. (MnTPPS_3_)_3.5_HSA was found to have a purity of 99.0% while (MnTPPS_3_)_6_HSA had a purity of 97.4%.

### 3.8. Stability of (MnTPPS_3_)_6_HSA

The stability of (MnTPPS_3_)_6_HSA against acid hydrolysis, demetalation, and trans-metalation was tested. Stock solution of (MnTPPS_3_)_6_HSA was mixed into test solutions comprising distilled water and HCl or stock solution of ZnCl_2_, with two replicates each. For acid-stress testing, the resulting solutions contained 0.25 mM (MnTPPS_3_)_6_HSA in either distilled water, 0.1 M (pH 1), or 0.001 M HCl (pH 3). For Zn-stress testing, the resulting solutions contained 0.25 mM (MnTPPS_3_)_6_HSA and 10 M ZnCl_2_ in either distilled water 0.1 M HCl, or 0.001 M HCl. Finally, to ensure that free Mn porphyrin and Mn could effectively bypass the filter, control samples were also prepared containing either 0.25 mM HSA with 1.5 mM MnCl_2_ or 0.25 mM HSA with 1.5 mM MnTPPS_3_NH_2_. Day 0 was prepared fresh as a solution of either 0.25 mM (MnTPPS_3_)_6_HSA in distilled water or 0.25 mM (MnTPPS_3_)_6_HSA and 10 M ZnCl_2_ in water. Solutions were stored in an incubator at 37 °C for 13 days before being removed and diluted with distilled water. These solutions were then filtered with 50 mL ultra spin filters (Amicon Ultra Regenerated cellulose 50 kDa MWCO) for 5 min at 4000 rpm to isolate free porphyrin and Mn into the filtrate. Spin filtration was repeated an additional 4 times after subsequent additions of 10 mM ammonium acetate to the retained (MnTPPS_3_)_6_HSA solutions. Resulting filtrates and retained solutions were weighed and their concentrations determined by the Thermo Scientific iCAP Pro ICP-OES measuring absorption at 257.610 nm and 213.856 nm. The resulting moles of Mn measured in each filtrate were compared to the corresponding moles of Mn measured in solutions retained behind the filter to determine the % of Mn removed from (MnTPPS_3_)_6_HSA solutions.

### 3.9. In-Vitro MRI of (MnTPPS_3_)_n_HSA

For in-vitro MRI measurements, 0.05 mM solutions of (MnTPPS_3_)_n_HSA (*n =* 3.5, 6, 9.5, 19) were dispensed into borosilicate glass tube phantoms with 4 mm inner diameters. Solutions were prepared by weighing out lyophilized HSA with concentrations based upon average *m*/*z* determined by MALDI-TOF/MS. Tubes were placed in a 7 × 5 ultem resin circular holder with each solution being at least 5 mm in depth. Imaging was performed on a research 1.5 T MRI scanner (Siemens Aera, Siemens Healthineers, Erlangen, Germany) in the UHN STTARR facility using two flat surface coils. Quantitative T_1_ relaxation times were measured using a two-dimensional (2D) inversion-recovery TSE sequence: inversion times (TI) = [25, 50, 75, 100, 200, 250, 500, 750, 1000, 1250, 1500, 1750, 2000, 2500] ms, repetition time (TR) = 3000 ms, echo time (TE) = 18.453 ms, field-of-view (FOV) = 60 mm, slice thickness (SL) = 2 mm, 0.4688 mm × 0.4688 mm in-plane resolution, and number of signal averages (NSA) = 1. Quantitative T_2_ relaxation times were measured using multi-echo SE sequence: 11 echoes with TE spacing = 12 ms, TR= 2500 ms. MRI data were transferred to an independent workstation for quantitative analysis using in-house software developed in MATLAB (v.9.10, MathWorks). Calculations of T_1_ and T_2_ relaxation times were performed on a pixel-by-pixel basis as described previously [42,43]. T_1_ and T_2_ were averaged over all pixels within an area of interest and reported as mean values and standard deviation.

### 3.10. In-Vivo MRI of Rat Injected with (MnTPPS_3_)_3.5_HSA and (MnTPPS_3_)_6_HSA

All animal procedures were performed in compliance with a protocol approved by the University Health Network’s Animal Resources Centre and associated animal care committee (protocol number: 6209). Two 8-weeks old Sprague Dawley female rats (Charles River Laboratories, Senneville, QC, Canada) were used as subjects. Rat 1 was injected via the tail vein with 0.65 mL of (MnTPPS_3_)_3.5_HSA at 0.01 mmol/kg of Mn and imaged over the course of 40 min. Rat 2 was injected via the tail vein with 1 mL of (MnTPPS_3_)_6_HSA at 0.02 mmol/kg of Mn and imaged over the course of 60 min. Imaging was performed on a research 1.5 T MRI scanner (Siemens Aera, Siemens Healthineers, Erlangen, Germany) in the UHN STTARR facility using two flat surface coils. Quantitative T_1_ relaxation times were measured using a three-dimensional (3D) T1-weighted VIBE sequence with variable flip angles: TR = 25 ms, TE = 3.52 ms, flip angles (FA) = [2, 10, 20, 25, 35], FOV = 20 cm, SL = 3 mm, 0.7821 mm × 0.781 mm in-plane resolution, and NSA = 1.

### 3.11. Labelled Collagen Stability Experiments

Acid-purified bovine type 1 collagen (Advanced Biomatrix, Carlsbad, CA, USA) at concentrations 3 and 10 mg mL^−1^ was dispensed (1 mL) into 14 mL round-bottom falcon tubes on ice. Next, 0.25 mL of cold stock solutions of one of MnP-NCS, APO-NH_2_, MnTPPS_4_, or MnP-NH_2_ was dispensed into tubes with collagen gel (*n =* 2) and mixed thoroughly. Next, to solidify gels, 0.25 mL of cold, freshly prepared 0.5 M Na_2_CO_3_ was added and mixed with collagen gels. Gels were then left in the fridge for 24 h at 4 °C to solidify before being transferred to 50 mL falcon tubes filled with PBS for an initial rinse before being left in an incubator at 37 °C for 24 h in 25 mL PBS. The PBS solution was then removed from gels, and then, the gels were rinsed 2 times with 40 mL PBS and centrifuged to remove remaining free porphyrin. These solutions were put aside for quantification of free porphyrin by UV–VIS and ICP. Then, 40 mL of PBS and the antibiotic streptomycin was added to collagen gels before being put back into the incubator at 37 °C. Rinse solutions of PBS containing free porphyrin were each weighed before measurement by UV–VIS (Figures S32–S34) to determine the amount of unbound collagen at day 0. Free porphyrin in sample solutions was measured periodically by UV–VIS (at 467 nm) over a month to determine the amount of porphyrin remaining inside collagen gels (Figures S35 and S36). After 556 days, sample solutions were measured by UV–VIS before collagen gels were removed for digestion for ICP analysis. For digestions, collagen samples were added to falcon tubes containing 2 mL 70% nitric acid and left overnight. Samples were subsequently sonicated for an hour at 40 °C before distilled water was added to dilute to 4% nitric acid and filtered through a 0.4 μm PTFE filter (EMD Millipore-Merck, Darmstadt, Germany). Digested collagen samples and their corresponding consolidated solutions composed of both initial rinse solutions and long-term leached porphyrin solutions were weighed and sent for ICP analysis on a Thermo Scientific iCAP Pro ICP OES to determine the moles of Mn to compare to results from UV–VIS. The estimated moles of porphyrin per mole of collagen or gram of collagen are provided in Figures S37 and S38 and were calculated from % binding determined from UV–VIS and ICP.

### 3.12. In-Vitro MRI of MnTPPS_3_ Labelled Collagen

The method of conjugating MnTPPS_3_NCS to acid soluble collagen and labeled hydrogel formation is outlined in the Supplementary. Gels were produced 5 and 60 days before MRI imaging on a clinical 3.0 T MRI scanner (Achieva 3.0T TX, Philips Medical Systems, Best, The Netherlands) using a 32-channel transmit/receive head coil. Quantitative T1 relaxation times were measured using a two-dimensional (2D) inversion-recovery TSE sequence: TI = [50, 100, 250, 500, 750, 1000, 1250, 1500, 2000, 2500] ms, TR = 3000 ms, TE = 18.4 ms, FOV = 12 cm, SL = 2 mm, 0.5 mm × 0.5 mm in-plane resolution, and NSA = 1. Quantitative T2 relaxation times were measured using a multi-echo SE sequence: 32 echoes with TE spacing = 7.626 ms, TR= 2000 ms.

## 4. Conclusions

This work demonstrates proof-of-principle for a pre-metalated conjugation platform that exploits a Gd-free T_1_ contrast agent for conjugating proteins or labeling materials to achieve specific, targeted imaging. The stability of our starting material, MnTPPS_3_NH_2_, surpassed any current Mn^2+^ or Gd-based contrast agents, resisting both demetalation and trans-metalation. Free from risk of demetalation, this compound was then converted to an electrophile, MnTPPS_3_NCS. Serving as a BFCA, MnTPPS_3_NCS achieved outstanding conjugation efficiency for the model human protein, plasma HSA, surpassing those recorded for previous chelates or fluorophores. The versatility and stability limits of MnTPPS_3_NCS conjugates were also tested with the labeling of collagen hydrogels. Labeled hydrogels produced substantial T_1_ signal enhancement, with the conjugated porphyrin remaining stable for over a year. The proposed MnTPPS_3_NCS platform offers a simple and effective technique for labeling proteins for targeted molecular imaging and for specialized MRI applications such as biomaterial tracking in regenerative applications.

## 5. Patents

We have a provisional patent on the work described herein: Cheng HLM, Vollett KDW, Szulc DA, Hong A. “Manganese bifunctional chelating agent conjugation platform for targeted MR imaging”. #GB2216233.3, 1 November 2022.

## Figures and Tables

**Table 1:** Bioconjugation reaction of MnTPPS_3_NCS with HSA at various stoichiometries.

	Eqv. Mn Added to HSA	Avg. *m*/*z*	Avg. Number of Mn/HSA	Tagging Efficiency (%)
Control (albumin only, in water)	0	66,500	--	--
MnTPPS_4_	5	66,600	0.09	1.8
	10	66,600	0.09	0.9
	20	67,300	0.8	4.0
	40	67,400	0.8	2.0
	80	67,400	0.8	1.0
MnTPPS_3_NCS	5	70,200	3.5	70
	10	72,500	6	60
	20	76,000	9.5	48
	40	85,500	19	48

## Data Availability

Data available upon request.

## Supplementary Materials

The supporting information can be downloaded at: https://www.mdpi.com/article/10.3390/ijms24119532/s1. References [18,35,41,44,45,46,47,48,49,50] are cited in the supplementary materials.

## References

[1] Lohrke J., Frenzel T., Endrikat J., Alves F.C., Grist T.M., Law M., Lee J.M., Leiner T., Li K.-C., Nikolaou K. (2016). 25 Years of Contrast-Enhanced MRI: Developments, Current Challenges and Future Perspectives. Adv. Ther..

[2] Aime S., Caravan P. (2009). Biodistribution of Gadolinium-Based Contrast Agents, Including Gadolinium Deposition. J. Magn. Reson. Imaging.

[3] (2016). Gadolinium. Meyler’s Side Effects of Drugs.

[4] Mathur M., Jones J.R., Weinreb J.C. (2020). Gadolinium Deposition and Nephrogenic Systemic Fibrosis: A Radiologist’s Primer. Radiographics.

[5] Li H., Meade T.J. (2019). Molecular Magnetic Resonance Imaging with Gd(III)-Based Contrast Agents: Challenges and Key Advances. J. Am. Chem. Soc..

[6] Unger E.C., Totty W.G., Neufeld D.M., Otsuka F.L., Murphy W.A., Welch M.S., Connett J.M., Philpott G.W. (1985). Magnetic Resonance Imaging Using Gadolinium Labeled Monoclonal Antibody. Investig. Radiol..

[7] Idée J.-M., Port M., Robic C., Medina C., Sabatou M., Corot C. (2009). Role of Thermodynamic and Kinetic Parameters in Gadolinium Chelate Stability. J. Magn. Reson. Imaging.

[8] Gale E.M., Atanasova I.P., Blasi F., Ay I., Caravan P. (2015). A Manganese Alternative to Gadolinium for MRI Contrast. J. Am. Chem. Soc..

[9] Tircsó G., Molnár E., Csupász T., Garda Z., Botár R., Kálmán F.K., Kovács Z., Brücher E., Tóth I., Sigel A., Freisinger E., Sigel R.K.O. (2021). Metal Ions in Bio-Imaging Techniques.

[10] Nwe K., Bernardo M., Regino C.A.S., Williams M., Brechbiel M.W. (2010). Comparison of MRI Properties between Derivatized DTPA and DOTA Gadolinium-Dendrimer Conjugates. Bioorg. Med. Chem..

[11] Nwe K., Xu H., Regino C.A.S., Bernardo M., Ileva L., Riffle L., Wong K.J., Brechbiel M.W. (2009). A New Approach in the Preparation of Dendrimer-Based Bifunctional Diethylenetriaminepentaacetic Acid MR Contrast Agent Derivatives. Bioconjug. Chem..

[12] Lattuada L., Barge A., Cravotto G., Giovenzana G.B., Tei L. (2011). The Synthesis and Application of Polyamino Polycarboxylic Bifunctional Chelating Agents. Chem. Soc. Rev..

[13] Cardinale J., Giammei C., Jouini N., Mindt T.L., Lewis J.S., Windhorst A.D., Zeglis B.M. (2019). Bioconjugation Methods for Radiopharmaceutical Chemistry BT—Radiopharmaceutical Chemistry.

[14] Brechbiel M.W. (2008). Bifunctional Chelates for Metal Nuclides. Q. J. Nucl. Med. Mol. Imaging.

[15] Geraldes C.F.G.C., Castro M.M.C.A., Peters J.A. (2021). Mn(III) Porphyrins as Potential MRI Contrast Agents for Diagnosis and MRI-Guided Therapy. Coord. Chem. Rev..

[16] Spang P., Herrmann C., Roesch F. (2016). Bifunctional Gallium-68 Chelators: Past, Present, and Future. Semin. Nucl. Med..

[17] Jing L., Liang X., Li X., Yang Y., Dai Z. (2013). Covalent Attachment of Mn-Porphyrin onto Doxorubicin-Loaded Poly(Lactic Acid) Nanoparticles for Potential Magnetic Resonance Imaging and PH-Sensitive Drug Delivery. Acta Biomater..

[18] Szulc D.A., Cheng H.-L.M. (2019). One-Step Labeling of Collagen Hydrogels with Polydopamine and Manganese Porphyrin for Non-Invasive Scaffold Tracking on Magnetic Resonance Imaging. Macromol. Biosci..

[19] Zhang Z., He R., Yan K., Guo Q., Lu Y., Wang X., Lei H., Li Z. (2009). Synthesis and in Vitro and in Vivo Evaluation of Manganese(III) Porphyrin-Dextran as a Novel MRI Contrast Agent. Bioorg. Med. Chem. Lett..

[20] Loai S., Szulc D.A., Cheng H.-L.M. (2022). Three-Dimensional Bioprinted MR-Trackable Regenerative Scaffold for Postimplantation Monitoring on T1-Weighted MRI. J. Magn. Reson. Imaging.

[21] Alhamami M., Cheng W., Lyu Y., Allen C., Zhang X.-A., Cheng H.-L. (2018). Manganese-Porphyrin-Enhanced MRI for the Detection of Cancer Cells: A Quantitative in Vitro Investigation with Multiple Clinical Subtypes of Breast Cancer. PLoS ONE.

[22] Venter A., Szulc D.A., Loai S., Ganesh T., Haedicke I.E., Cheng H.-L.M. (2018). A Manganese Porphyrin-Based T1 Contrast Agent for Cellular MR Imaging of Human Embryonic Stem Cells. Sci. Rep..

[23] Lyon R.C., Faustino P.J., Cohen J.S., Katz A., Mornex F., Colcher D., Baglin C., Koenig S.H., Hambright P. (1987). Tissue Distribution and Stability of Metalloporphyrin MRI Contrast Agents. Magn. Reson. Med..

[24] Fiel R.J., Musser D.A., Mark E.H., Mazurchuk R., Alletto J.J. (1990). A Comparative Study of Manganese Meso-Sulfonatophenyl Porphyrins: Contrast-Enhancing Agents for Tumors. Magn. Reson. Imaging.

[25] Szulc D.A., Ahmadipour M., Aoki F.G., Waddell T.K., Karoubi G., Cheng H.-L.M. (2020). MRI Method for Labeling and Imaging Decellularized Extracellular Matrix Scaffolds for Tissue Engineering. Magn. Reson. Med..

[26] Wedeking P., Kumar K., Tweedle M.F. (1992). Dissociation of Gadolinium Chelates in Mice: Relationship to Chemical Characteristics. Magn. Reson. Imaging.

[27] Frenzel T., Lengsfeld P., Schirmer H., Hütter J., Weinmann H.-J. (2008). Stability of Gadolinium-Based Magnetic Resonance Imaging Contrast Agents in Human Serum at 37 Degrees C. Investig. Radiol..

[28] Sherry A.D., Caravan P., Lenkinski R.E. (2009). Primer on Gadolinium Chemistry. J. Magn. Reson. Imaging.

[29] Morcos S.K. (2008). Extracellular Gadolinium Contrast Agents: Differences in Stability. Eur. J. Radiol..

[30] Sarka L., Burai L., Király R., Zékány L., Brücher E. (2002). Studies on the Kinetic Stabilities of the Gd^3+^ Complexes Formed with the N-Mono(Methylamide), N′-Mono(Methylamide) and N,N″-Bis(Methylamide) Derivatives of Diethylenetriamine-N,N,N′,N″,N″-Pentaacetic Acid. J. Inorg. Biochem..

[31] Ndiaye D., Sy M., Pallier A., Même S., de Silva I., Lacerda S., Nonat A.M., Charbonnière L.J., Tóth É., Ndiaye D. (2020). Unprecedented Kinetic Inertness for a Mn^2+^-Bispidine Chelate: A Novel Structural Entry for Mn^2+^-Based Imaging Agents. Angew. Chem. Int. Ed..

[32] Chilla S.N.M., Laurent S., Vander Elst L., Muller R.N. (2014). Synthesis and Characterization of a New Lanthanide Based MRI Contrast Agent, Potential and Versatile Tracer for Multimodal Imaging. Tetrahedron.

[33] Tan S., Yang H., Xue S., Qiao J., Salarian M., Hekmatyar K., Meng Y., Mukkavilli R., Pu F., Odubade O.Y. (2020). Chemokine Receptor 4 Targeted Protein MRI Contrast Agent for Early Detection of Liver Metastases. Sci. Adv..

[34] Dai L., Jones C.M., Chan W.T.K., Pham T.A., Ling X., Gale E.M., Rotile N.J., Tai W.C.-S., Anderson C.J., Caravan P. (2018). Chiral DOTA Chelators as an Improved Platform for Biomedical Imaging and Therapy Applications. Nat. Commun..

[35] Nwe K., Milenic D., Bryant L.H., Regino C.A.S., Brechbiel M.W. (2011). Preparation, Characterization and in Vivo Assessment of Gd-Albumin and Gd-Dendrimer Conjugates as Intravascular Contrast-Enhancing Agents for MRI. J. Inorg. Biochem..

[36] Fisher M.J., Williamson D.J., Burslem G.M., Plante J.P., Manfield I.W., Tiede C., Ault J.R., Stockley P.G., Plein S., Maqbool A. (2015). Trivalent Gd-DOTA Reagents for Modification of Proteins. RSC Adv..

[37] Frullano L., Caravan P. (2011). Strategies for the Preparation of Bifunctional Gadolinium(III) Chelators. Curr. Org. Synth..

[38] Franano F.N., Edwards W.B., Welch M.J., Brechbiel M.W., Gansow O.A., Duncan J.R. (1995). Biodistribution and Metabolism of Targeted and Nontargeted Protein-Chelate-Gadolinium Complexes: Evidence for Gadolinium Dissociation in Vitro and in Vivo. Magn. Reson. Imaging.

[39] Doyle A.D. (2018). Fluorescent Labeling of Rat-Tail Collagen for 3D Fluorescence Imaging. Bio-Protocol.

[40] Schneider E., Brendle K., Jäger P., Weis P., Kappes M.M. (2018). Ion Mobility Measurements of Multianionic Metalloporphyrin Dimers: Structural Changes Induced by Countercation Exchange. J. Am. Soc. Mass Spectrom..

[41] Fodor M.A., Szabó P., Lendvay G., Horváth O. (2022). Characterization of the UV-Visible Absorption Spectra of Manganese(III) Porphyrins with Time-Dependent Density Functional Theory Calculations. Z. Phys. Chem..

[42] Cheng H.L., Wright G.A. (2006). Rapid High-Resolution T(1) Mapping by Variable Flip Angles: Accurate and Precise Measurements in the Presence of Radiofrequency Field Inhomogeneity. Magn. Reson. Med..

[43] Beaumont M., Odame I., Babyn P.S., Vidarsson L., Kirby-Allen M., Cheng H.-L.M. (2009). Accurate Liver T2 Measurement of Iron Overload: A Simulations Investigation and in Vivo Study. J. Magn. Reson. Imaging.

[44] Mcdonagh P., Williams K. (1984). The Preparation and Use of Fluorescent-Protein Conjugates for Microvascular Research. Microvasc. Res..

[45] Jakubowski N., Waentig L., Hayen H., Venkatachalam A., Von Bohlen A., Roos P., Manz M. (2008). Labelling of proteins with 2-(4-isothiocyanatobenzyl)-1,4,7,10-tetraazacyclododecane-1,4,7,10-tetraacetic acid and lanthanidesand detection by ICP-MS. J. Anal. At. Spectrom..

[46] Caviglioli G., Parodi B., Cafaggi S., Russo E., Cirrincione P., Chinol M., Paganelli G. (2013). A Conjugate of Human Albumin and 2-(4-Isothiocyanatobenzyl)-1,4,7,10-tetraazacyclododecane-1,4,7,10-tetraacetic Acid Useful for the Localization of Radionuclides for Diagnostic and Therapeutic Purposes.

[47] Cassells I., Ahenkorah A., Burgoyne A., Van de Voorde M., Deroose R., Cardinaels T., Bormans G., Ooms M., Cleeren F. (2021). Radiolabeling of Human Serum Albumin With Terbium-161 Using Mild Conditions and Evaluation of in vivo Stability. Front. Med..

[48] Ogan M., Schmiedl U., Moseley M., Grodd W., Paajanen H., Brasch R. (1990). Albumin labeled with Gd-DTPA. An intravascular contrast-enhancing agent for magnetic resonance blood pool and perfusion imaging. Acta Radiol. Suppl..

[49] Maisano F., Gozzini L. (2000). Process for the Conjugation of Chelants with Molecules Containing Amino Groups.

[50] Kundu A., Peterlik H., Krssak M., Bytzek A., Pashkunova I., Arion V., Helbich T., Keppler B. (2011). Strategies for the covalent conjugation of a bifunctional chelating agent to albumin: Synthesis and characterization of potential MRI contrast agents. J. Inorg. Biochem..

