# Supplementary material for: A Manganese Porphyrin Platform for the Design and Synthesis of Molecular and Targeted MRI Contrast Agents
Source: Int J Mol Sci. 2023 May 31;24(11):9532. doi: 10.3390/ijms24119532 (PMC10253719; doi:10.3390/ijms24119532)
Supplement: Supplementary file 1 [file ijms-24-09532-s001.zip › ijms-2392838-supplementary.pdf]

Supporting Information

A manganese porphyrin platform for the design and synthesis of molecular and targeted MRI contrast agents

Kyle D.W. Vollett, BSc[1,2] ǂ, Daniel A. Szulc, PhD[1,2] ǂ, Hai-Ling Margaret Cheng, PhD, PEng[1-3]

ǂ Co-authorship

[1] Institute of Biomedical Engineering, University of Toronto, Canada

[2] Translational Biology & Engineering Program, Ted Rogers Centre for Heart Research, Toronto, Canada

[3] The Edward S. Rogers Sr. Department of Electrical and Computer Engineering, University of Toronto, Canada

Metalation of Amine Manganese (III) Porphyrin ($MnTPPS_3NH_2$, $ZnTPPS_3NH_2$)

The procedures for the insertion of manganese(III) into 5-(4-aminophenyl)-10,15,20-(tri-4-sulfonatophenyl)porphyrin was adopted from previously described methods [18]. In brief the monoamine porphyrin 5-(4-aminophenyl)-10,15,20-(tri-4-sulfonatophenyl)porphyrin arrived as a ammonium salt and was exchanged with sodium by running through a pre-loaded amberlite IR120 ion exchange resin to form the trisodium salt ($TPPS_3NH_2$). The eluted porphyrin solution was then immediately dried by lyophilization and stored for future use. Metalation was performed as outlined in **scheme s1**, where a 1 g (1.03 mmol) fraction of $TPPS_3NH_2$ and 8g (40 mmol) $MnCl{\cdot}H_2O$ was added to a round bottom flask with 58 mL of dimethylformamide and dissolved with rigorous stirring. Following this, N,N-Diisopropylethylamine (DIPEA) was added dropwise to the reaction solution before it was put under reflux at 135 ^oC for 3 hours. The resulting manganese (III) 5-(4-aminophenyl)-10,15,20-(tri-4-sulfonatophenyl) chloride porphyrin ($MnTPPS_3NH_2$) solution was subjected to roto-evaporation to remove DMF and then dissolved in minimal water. Excess salt was removed by running the porphyrin solution through a manually packed C18 column with 100% H_2O and compressed air to speed up elution. $MnTPPS_3NH_2$ was retained in the column allowing for 10 column volume flushes of water to remove most of the salt. $MnTPPS_3NH_2$ was then eluted with 100% methanol, which was removed with roto-evaporator and redissolved in a minimal amount of water. This $MnTPPS_3NH_2$ solution was loaded into dialysis tubing (MWCO: 1 kD) and left in deionized water with two water exchanges over 48 hours. Exchange of Mn^{2+} for Na^+ was performed with ion exchange using a self-packed amberlite IR120 resin column and deionized water for elution. Ion exchange column was previously loaded with 1 M NaOH. The resulting $MnTPPS_3NH_2$ porphyrin was then dried by lyophilization as a green powder. MS (ESI) *m/z* calculated for $[M_2\text{-}6H]^{4-}$ $((C_{44}H_{26}N_5O_9S_3Mn)_2)^{4-}$: 459.5142. Found: 459.5137. $\varepsilon = 74{,}529\ M^{-1}cm^{-1}$ Max at 467 nm.

For $ZnTPPS_3NH_2$ porphyrin. MS (ESI) *m/z* calculated for $[M_2\text{-}6H]^{2-}$ $((C_{44}H_{27}N_5O_9S_3Zn)_2)^{2-}$: 464.5137. Found: 464.5135. $\varepsilon = 243{,}300\ M^{-1}cm^{-1}$ Max at 421 nm.

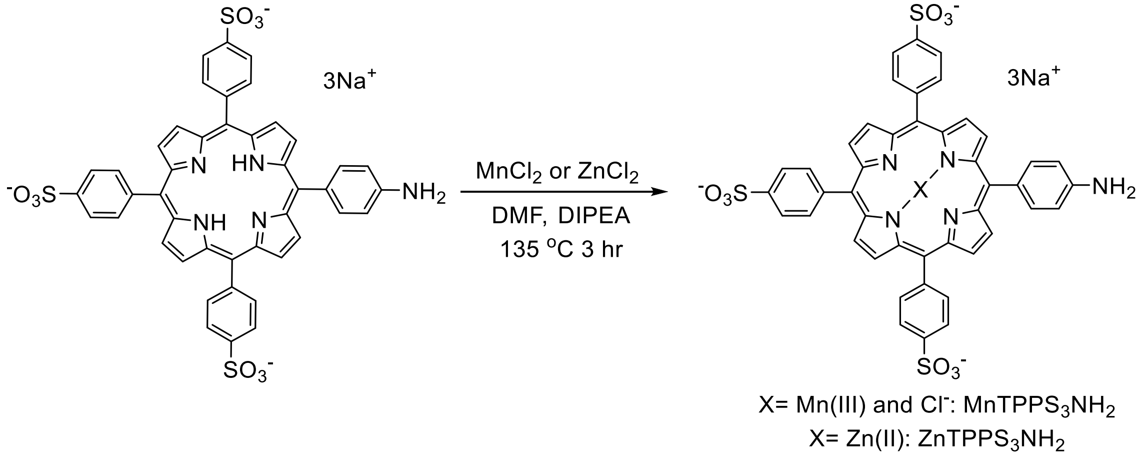

Scheme s1. Insertion of Mn(III) or Zn(II) into $TPPS_3NH_2$.

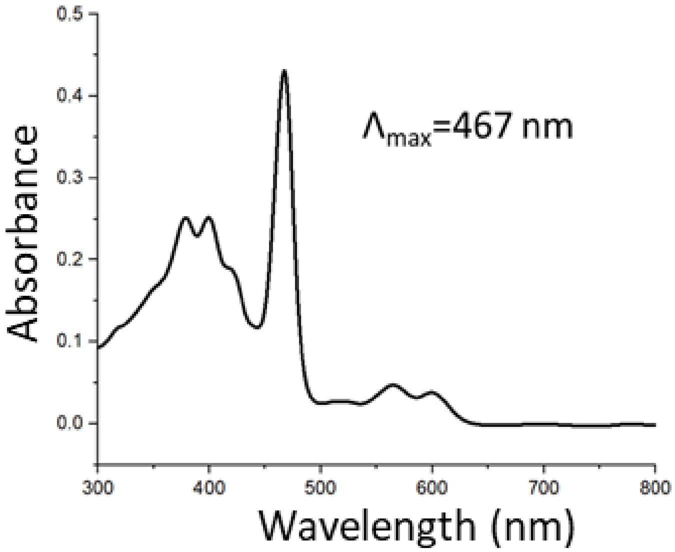

Figure s1. UV-VIS spectrum of $MnTPPS_3NH_2$ with Mn(III) oxidation state confirmed by Soret band[2] at λ_{max} = 467 nm (ε = 74,529 $M^{-1}cm^{-1}$) measured in PBS at 25 ^oC.

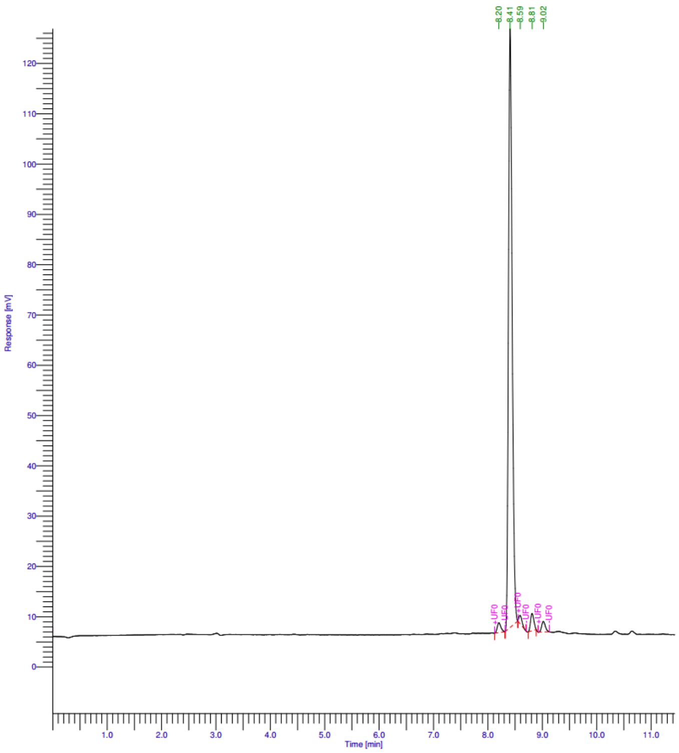

Figure s2. HPLC spectra of $MnTPPS_3NH_2$ were recorded using a **PerkinElmer Series 200 system** with UV/Vis detectors recording at 467 nm and using an acetonitrile and 10 mM ammonium acetate (NH4OAc) gradient mix. Elution occurred at 8.41 minutes with 93.37% purity.

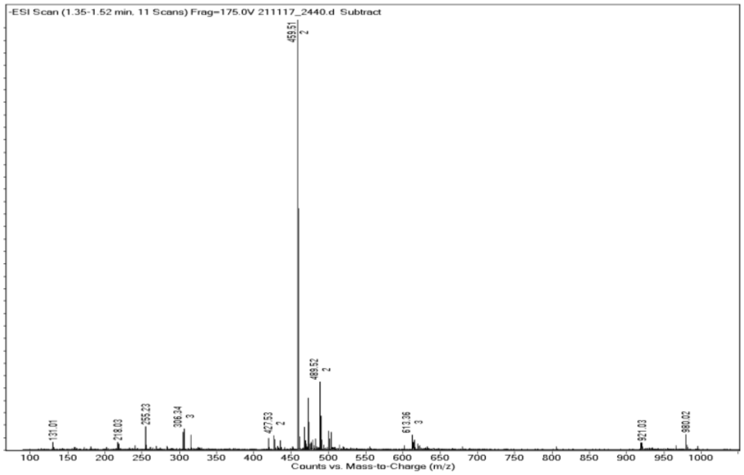

Target Ion Species

Ion Species	*m/z*	*Ionic Formula*
(M-2H)-2	459.5137	C44 H26 Mn N5 O9 S3

MFG Calculator Results

Target m/z	*Ionic Formula*	*Calc m/z*	*+/- (mDa)*	*+/- (ppm)*	*MFG Score*
459.5137	C44 H26 Mn N5 O9 S3	459.5142	-0.5	-1.1	97.89
459.5137	C45 H22 Mn N9 O5 S3	459.5149	-1.2	-2.6	96.73
459.5137	C56 H22 Mn N3 O2 S3	459.5133	0.4	0.9	96.51
459.5137	C49 H22 Mn N9 S4	459.5136	0.1	0.2	94.98
459.5137	C48 H26 Mn N5 O4 S4	459.5130	0.7	1.5	92.85
459.5137	C53 H26 Mn N3 O2 S4	459.5150	-1.3	-2.8	91.01
459.5137	C52 H18 Mn N9 S3	459.5119	1.8	3.9	91.00
459.5137	C47 H30 Mn N O8 S4	459.5123	1.4	3.0	88.63
459.5137	C61 H22 Mn N S3	459.5153	-1.6	-3.5	88.42
459.5137	C49 H26 Mn N3 O7 S3	459.5162	-2.5	-5.4	86.77

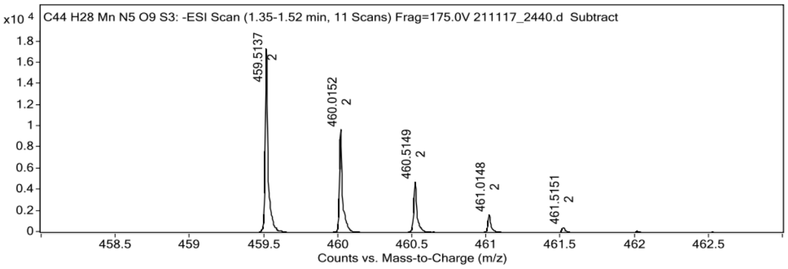

Predicted Isotope Match Table

Isotope	*m/z*	Calc m/z	Diff (mDa)	Abund (%)	Calc Abund (%)	+/-
1	459.5137	459.5142	-0.5	100.0	100.0	0.0
2	460.0152	460.0157	-0.5	55.4	52.4	-3.0
3	460.5149	460.5148	0.1	27.3	28.7	1.4
4	461.0148	461.0151	-0.3	9.7	10.2	0.5
5	461.5151	461.5148	0.3	2.9	3.2	0.3

Figure s3. Mass Spectrum obtained for $MnTPPS_3NH_2$ with an Agilent 6538 Q-TOF system in ESI MS Negative mode. MS (ESI) *m/z* calculated for $[M_2\text{-}6H]^{4-}$ $((C_{44}H_{26}N_5O_9S_3Mn)_2)^{4-}$: 459.5142. Found: 459.5137. MS (ESI) *m/z* calculated for $[M_2\text{-}4H]^{3-}$ $((C_4H_{27}N_5O_9S_3Mn)_2)^{3-}$: 613.36. Found: 613.36. Is consistent with dimers formed by sulfonatophenyl metalloporphyrins.

Stability of acid-stressed Gadovist. The stability of Gadovist against de-metalation was tested in PBS and 0.1 M HCl at 37 ºC. Stock solution of Gadovist was mixed with HCl and water in two replicates each with resulting solutions of 0.25 mM Gadovist. Solutions were stored in an incubator at 37 ºC for either 6, 24 and 48 hours. Samples were then diluted in 10 % acetonitrile and immediately assessed by Waters ACQUITY H-class UHPLC system with PDA detector and single quadrupole MS detector at the University of Toronto Department of Chemistry ANALEST facility. Relative concentrations of Gadovist and the Gd free chelate species was estimated by the change in peak areas relative to stock solution detected at 274 nm eluting at 30 and 40 seconds, respectively. Gadovist species identified with Waters ACQUITY single quadrupole MS detector system in ESI MS Positive mode with MS (ESI) *m/z* calcd for $[M]^+$ $C_{18}H_{35}N_4O_9^+$: 451.24. Found: 451.42 for Gd free Gadovist chelate. And MS (ESI) *m/z* calcd for $[M]^+$ $C_{18}H_{35}GdN_4O_9^+$: 606.14. Found: 606.35 for intact Gadovist.

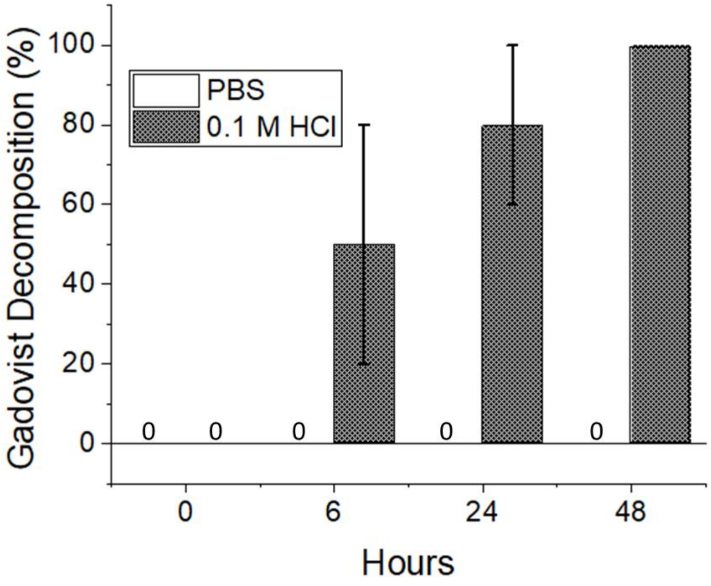

Figure s4. Gadovist in 0.1 M HCl solution over 48 hours. Percentage demetalation of Gadovist measured by the relative concentration of metal-free BT-DO3A chelate.

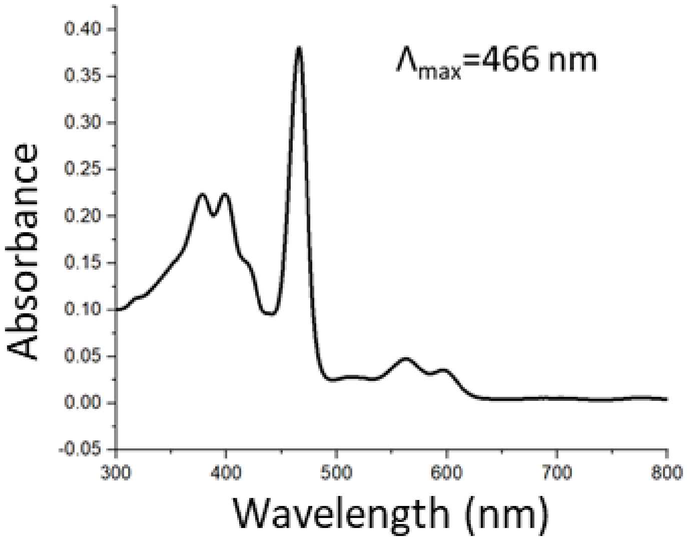

Figure s5. UV spectrum of $MnTPPS_3NCS$ (λ_{max} = 466 nm, ε = 89,196 $M^{-1}cm^{-1}$) measured in PBS at 25 ^{o}C.

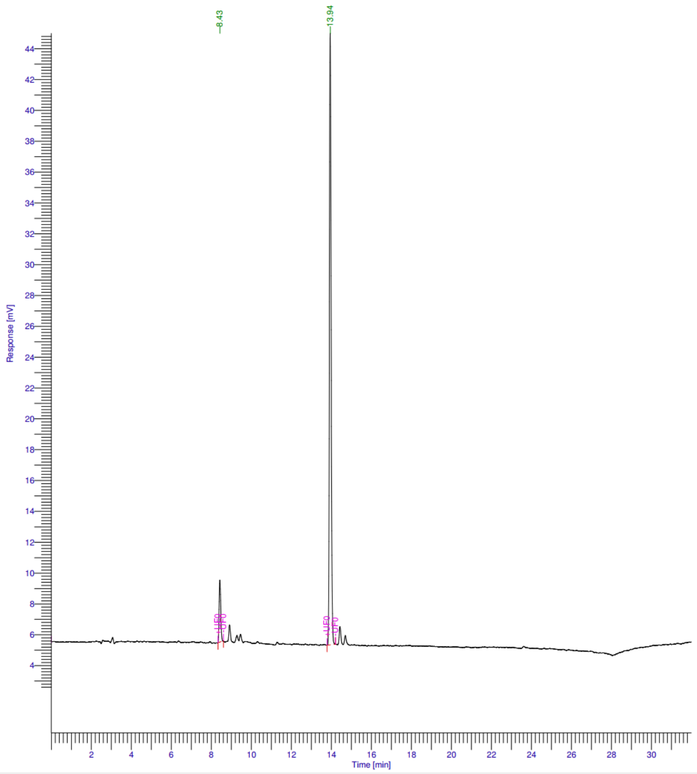

Figure s6. HPLC spectra of $MnTPPS_3NCS$ were recorded using a **PerkinElmer Series 200 system** with UV/Vis detectors recording at 467 nm and using an acetonitrile and 10 mM ammonium acetate (NH_4OAc) gradient mix. Elution occurred at 13.94 minutes with 91.8% purity.

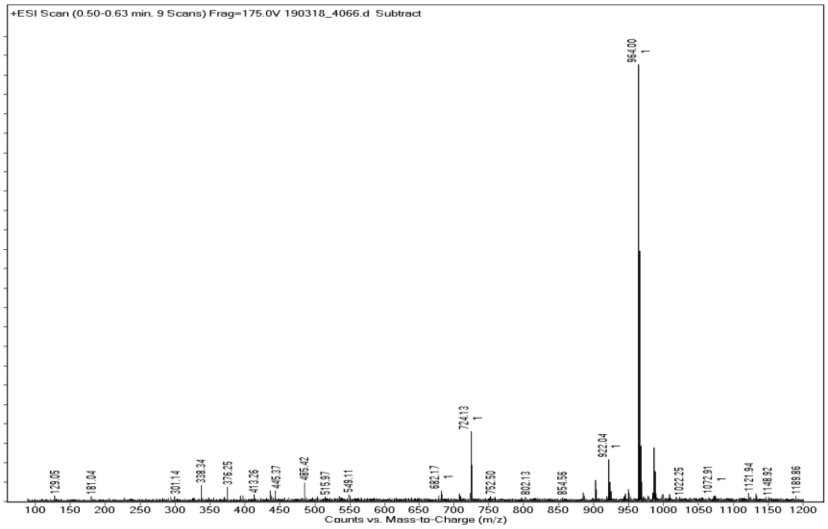

Figure s7. Mass Spectrum obtained for MnTPPS$_3$NCS with an Agilent 6538 Q-TOF system in ESI MS Positive mode. MS (ESI) *m/z* calcd for $[M]^+$ $C_{45}H_{27}N_5O_9S_4Mn^+$: 964.01. Found: 964.00.

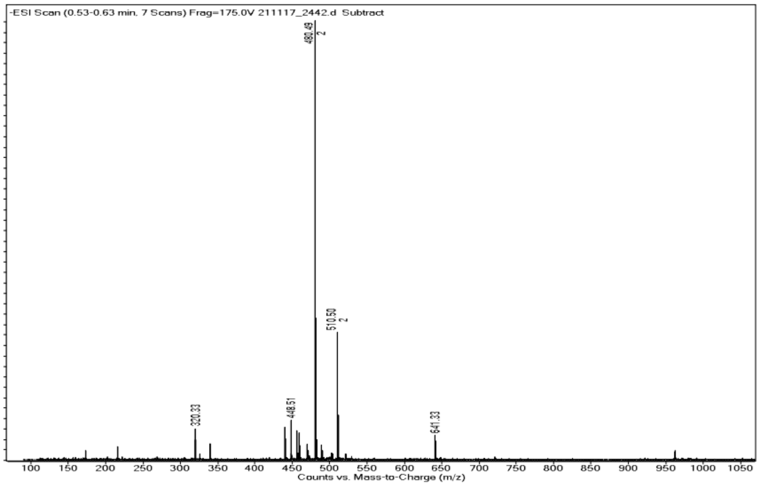

Target Ion Species

Ion Species	m/z	Ionic Formula
(M-2H)-2	480.4918	C45 H24 Mn N5 O9 S4

MFG Calculator Results

Target m/z	Ionic Formula	Calc m/z	+/- (mDa)	+/- (ppm)	MFG Score
480.4918	C49 H16 Mn N9 O5 S3	480.4914	0.4	0.8	98.88
480.4918	C53 H20 Mn N3 O7 S3	480.4927	-0.9	-1.9	97.34
480.4918	C45 H24 Mn N5 O9 S4	480.4924	-0.6	-1.2	96.90
480.4918	C48 H20 Mn N5 O9 S3	480.4907	1.1	2.3	96.86
480.4918	C57 H20 Mn N3 O2 S4	480.4915	0.3	0.6	96.61
480.4918	C46 H20 Mn N9 O5 S4	480.4931	-1.3	-2.7	95.92
480.4918	C54 H16 Mn N7 O3 S3	480.4934	-1.6	-3.3	93.34
480.4918	C53 H16 Mn N9 S4	480.4901	1.7	3.5	91.39
480.4918	C40 H24 Mn N7 O11 S4	480.4904	1.4	2.9	89.30
480.4918	C60 H16 Mn N3 O2 S3	480.4898	2.0	4.2	89.02
480.5895	C60 H44 Mn N S4	480.5874	2.1	4.4	83.25
480.5895	C56 H44 Mn N O5 S3	480.5887	0.8	1.7	82.31
480.5895	C49 H44 Mn N7 O3 S4	480.5890	0.5	1.0	81.24
480.5895	C57 H40 Mn N5 O S3	480.5893	0.2	0.4	81.20
480.5895	C52 H40 Mn N7 O3 S3	480.5873	2.2	4.6	80.01
480.5895	C51 H44 Mn N3 O7 S3	480.5866	2.9	6.0	75.43
480.5895	C53 H48 Mn N O5 S4	480.5903	-0.8	-1.7	74.40
480.5895	C54 H44 Mn N5 O S4	480.5910	-1.5	-3.1	70.96
480.5895	C49 H48 Mn N O10 S3	480.5916	-2.1	-4.4	60.38
480.5895	C50 H44 Mn N5 O6 S3	480.5923	-2.8	-5.8	56.51

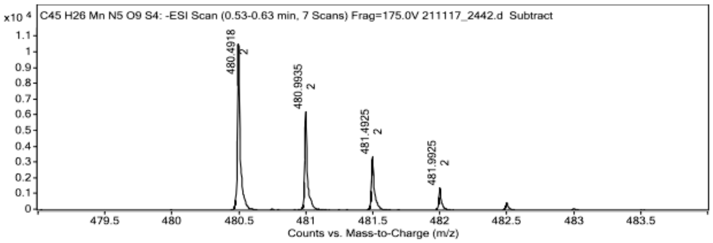

Predicted Isotope Match Table

Isotope	m/z	Calc m/z	Diff (mDa)	Abund (%)	Calc Abund (%)	+/-
1	480.4918	480.4924	-0.6	100.0	100.0	0.0
2	480.9935	480.9939	-0.4	58.0	54.3	-3.7
3	481.4925	481.4927	-0.2	31.5	34.2	2.7
4	481.9925	481.9931	-0.6	13.0	13.1	0.1

:ope	m/z	Calc m/z	Diff (mDa)	Abund (%)	Calc Abund (%)	+/-
5	482.4933	482.4925	0.8	4.5	4.7	0.2

Figure s8. Mass Spectrum obtained for MnTPPS$_3$NCS with an Agilent 6538 Q-TOF system in ESI MS Negative mode. MS (ESI) *m/z* calcd for $[M_2\text{-}6H]^{4-}$ $((C_{45}H_{24}N_5O_9S_4Mn)_2)^{4-}$: 480.4924. Found: 480.4918 Is consistent with dimers formed by sulfonatophenyl metalloporphyrins.

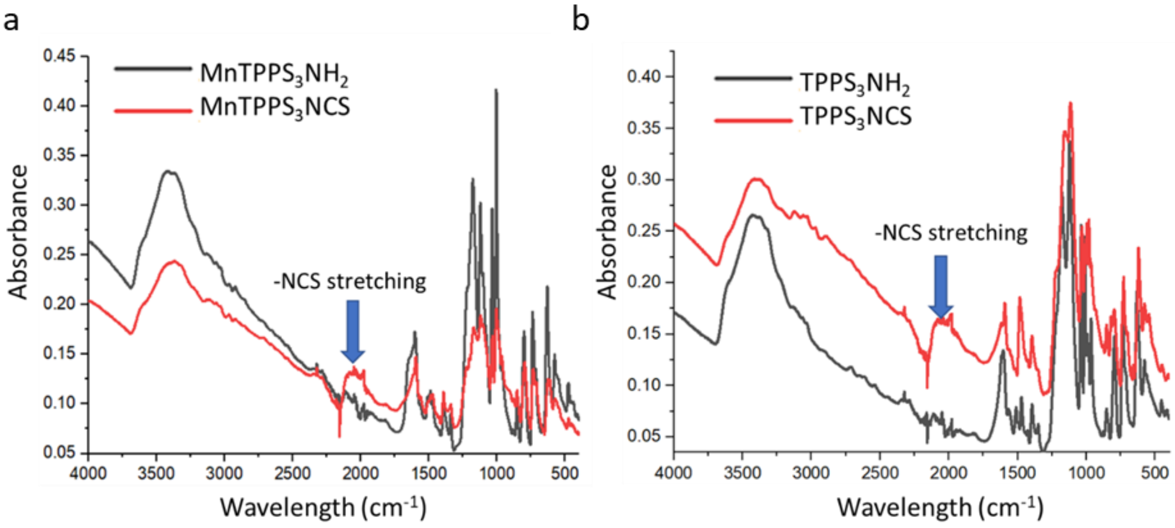

Figure s9. Fourier transform infrared spectroscopy (FTIR-ATR, Thermo Scientific Nicolet iS50) of (a) $MnTPPS_3NH_2$ and (b) $TPPS_3NCS$ with their respective amine precursors. Emphasis on absorption peak at 2050 cm^{-1} associated with isothiocyanate stretching.

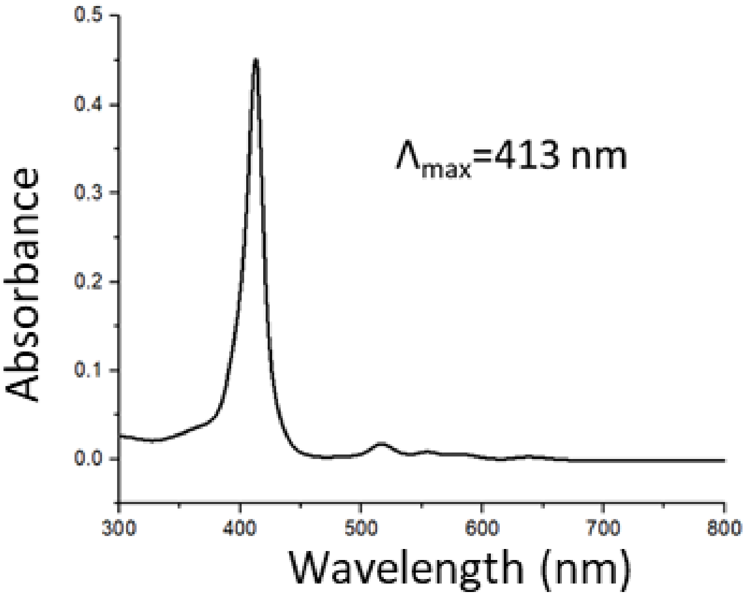

Figure s10. UV spectrum of $TPPS_3NCS$ (λ_{max} = 413 nm, ε = 224,272 $M^{-1}cm^{-1}$) measured in PBS at 25 ^{o}C.

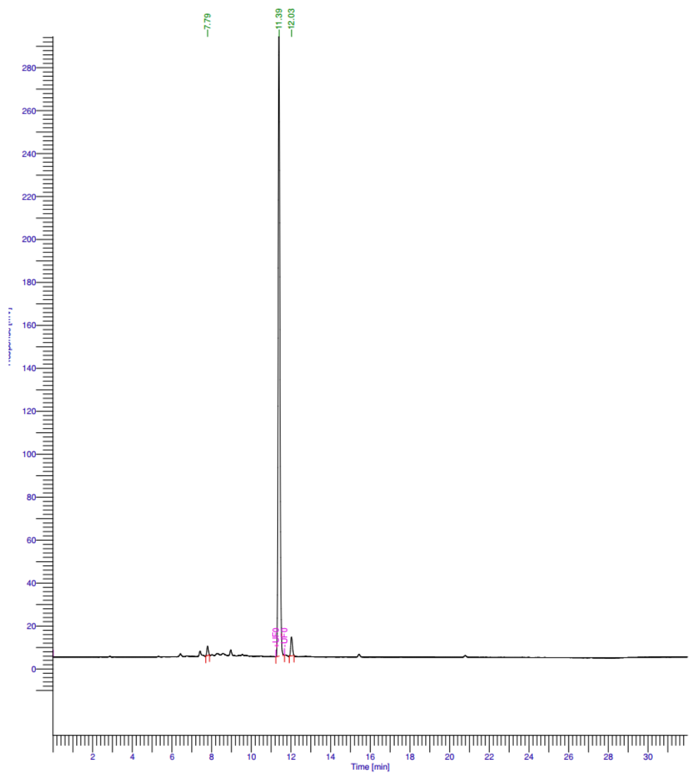

Figure s11. HPLC spectra of $TPPS_3NCS$ were recorded using a **PerkinElmer Series 200 system** with UV/Vis detectors recording at 419 nm and using an acetonitrile and 10 mM ammonium acetate (NH_4OAc) gradient mix. Elution occurred at 11.39 minutes with 94.25% purity.

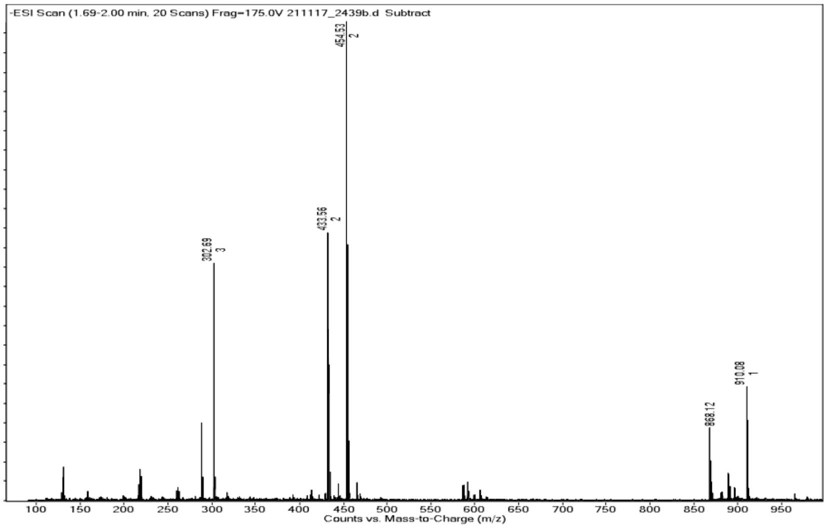

Target Ion Species

Ion Species	m/z	Ionic Formula
(M-2H)-2	454.5347	C45 H27 N5 O9 S4

MFG Calculator Results

Target m/z	Ionic Formula	Calc m/z	+/- (mDa)	+/- (ppm)	DBE	MFG Score
454.5347	C45 H27 N5 O9 S4	454.5351	-0.4	-0.9	34.0	98.83
454.5347	C46 H23 N9 O5 S4	454.5358	-1.1	-2.4	39.0	97.10
454.5347	C57 H23 N3 O2 S4	454.5342	0.5	1.1	47.0	94.58
454.5347	C53 H19 N9 S4	454.5329	1.8	4.0	48.0	88.51
454.5347	C50 H27 N3 O7 S4	454.5371	-2.4	-5.3	38.0	87.05
454.5347	C52 H23 N5 O4 S4	454.5322	2.5	5.5	43.0	84.35
454.5347	C51 H23 N7 O3 S4	454.5378	-3.1	-6.8	43.0	80.84
454.5347	C51 H27 N O8 S4	454.5315	3.2	7.0	38.0	79.37

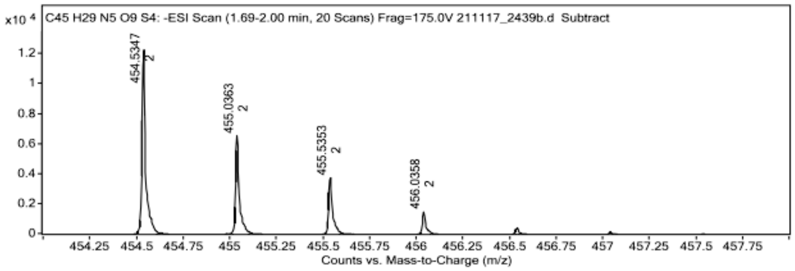

Predicted Isotope Match Table

Isotope	m/z	Calc m/z	Diff (mDa)	Abund (%)	Calc Abund (%)	+/-
1	454.5347	454.5351	-0.4	100.0	100.0	0.0
2	455.0363	455.0366	-0.3	53.2	54.3	1.1
3	455.5353	455.5354	-0.1	31.0	34.2	3.2
4	456.0358	456.0358	0.0	12.6	13.1	0.5
5	456.5351	456.5352	-0.1	3.7	4.7	1.0

Figure s12. Mass Spectrum obtained for $TPPS_3NCS$ with an Agilent 6538 Q-TOF system in ESI MS Negative mode. MS (ESI) *m/z* calcd for $[M-2H]^{2-}$ $C_{45}H_{27}N_5O_9S_4^{-2}$: 454.5351. Found: 454.5347. Calcd for $[M-3H]^{3-}$ $C_{45}H_{26}N_5O_9S_4^{-3}$: 306.69. Found: 302.69. Calcd for $[M]^-$ $C_{45}H_{28}N_5O_9S_4^-$: 910.08. Found: 910.08.

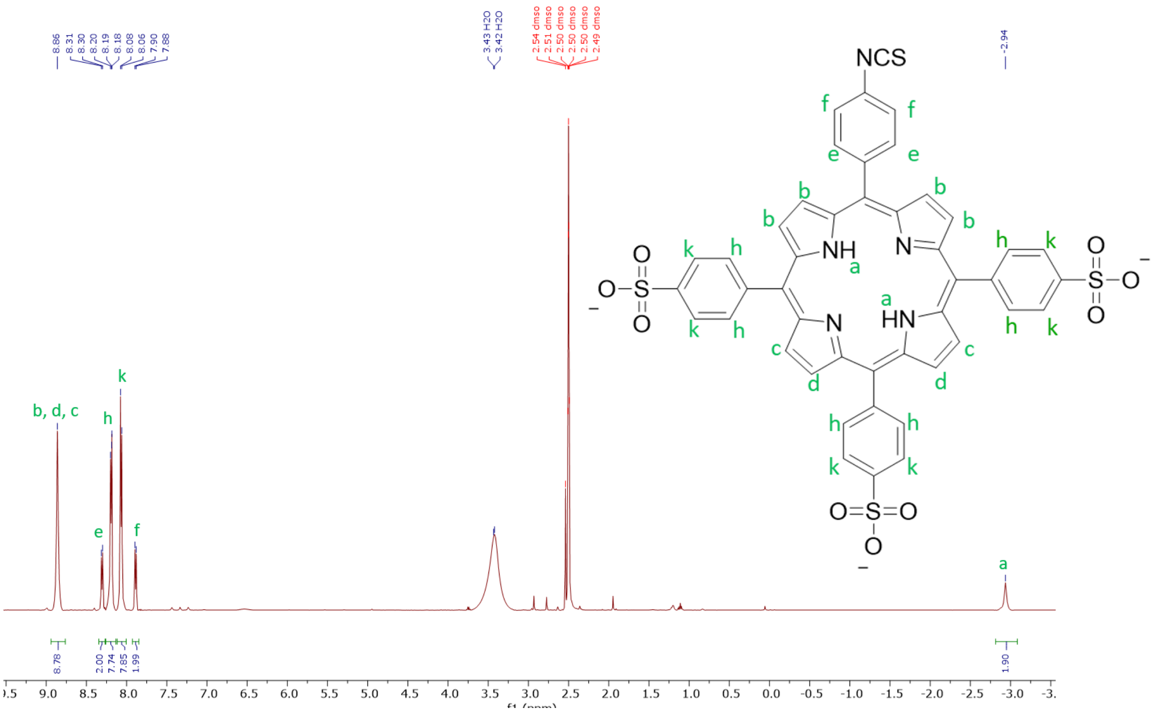

Figure s13. H^1NMR spectrum of $TPPS_3NCS$ acquired on Bruker US 500 MHz system. **^{1}H NMR (500 MHz, DMSO-d_6)** δ (ppm) 8.86 (m, 8H, β-pyrrole), 8.30 (m , J= 7.9 Hz, 2H), 8.19 (m, 6H), 8.06 (m 6H), 7.89 (d, 2H, J=7.9 Hz), -2.94 (s, 2H).

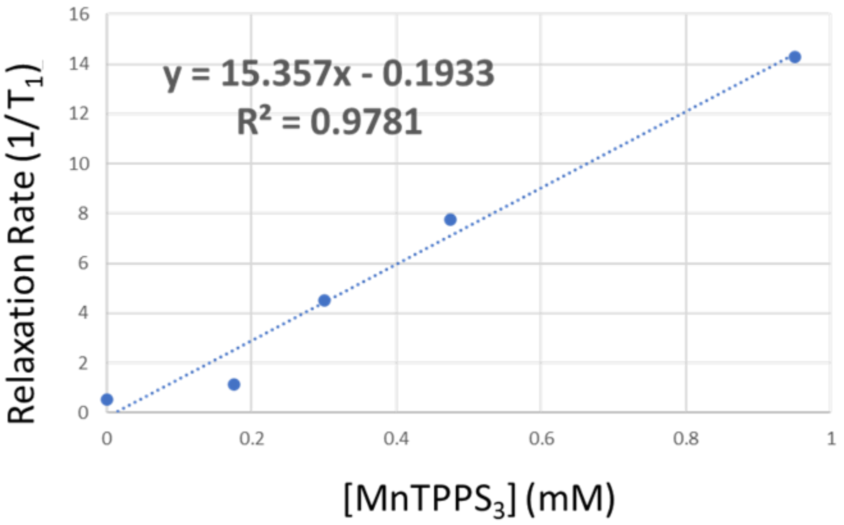

Figure s14. Relaxivity measurement of $(MnTPPS_3)_{3.5}HSA$, $(MnTPPS_3)_6HSA$, $(MnTPPS_3)_{9.5}HSA$, and $(MnTPPS_3)_{19}HSA$ all at 0.05 M. The resulting [$MnTPPS_3$] concentration is based upon the average # of Mn/HSA determined by MALDI-TOF/MS. For example, the concentration of $(MnTPPS_3)_6HSA$ is 0.05 M; the concentration of $MnTPPS_3$ is 6×0.05 M.

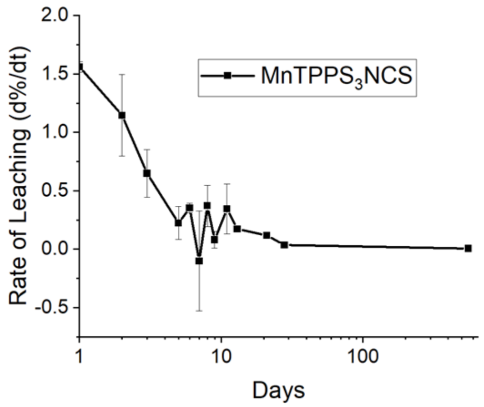

Figure s15. Rate of release of $MnTPPS_3NCS$-labeled collagen hydrogels over 556 days after labeling. Indicates that free $MnTPPS_3NH_2$ is initially leaching from gels before stabilizing at around 5 days.

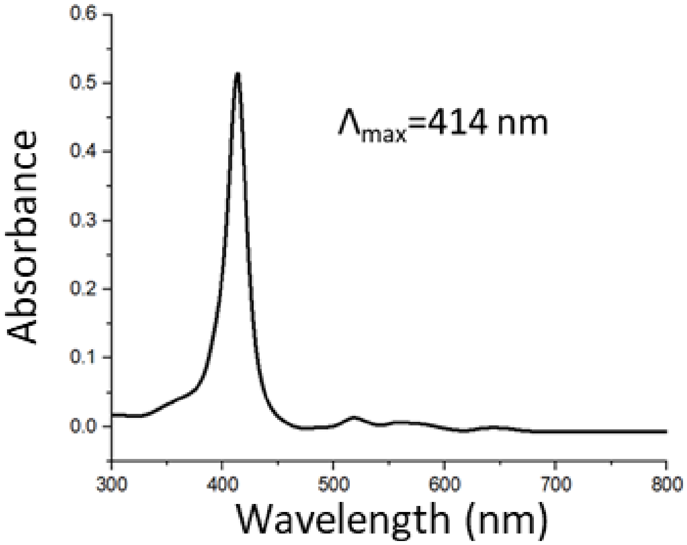

Figure s16. UV spectrum of $TPPS_3NH_2$ (λ_{max} = 414 nm, ε = 231,885 $M^{-1}cm^{-1}$) measured in PBS at 25 ^{o}C.

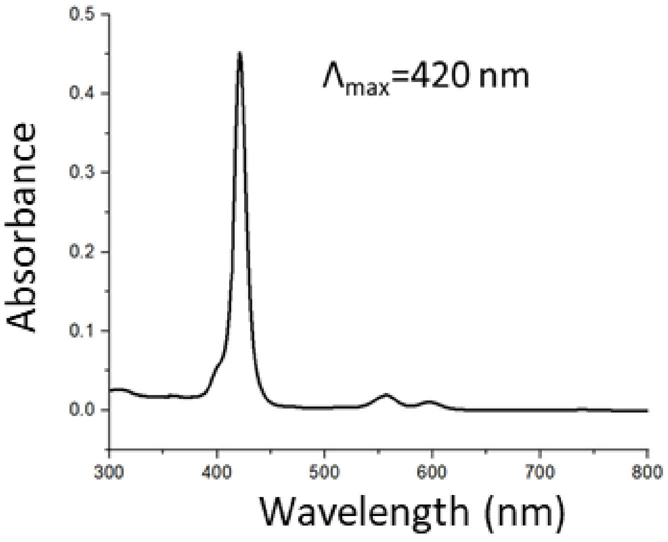

Figure s17. UV spectrum of $ZnTPPS_3NH_2$ (λ_{max} = 420 nm, ε = 243,300 $M^{-1}cm^{-1}$) measured in PBS at 25 ºC.

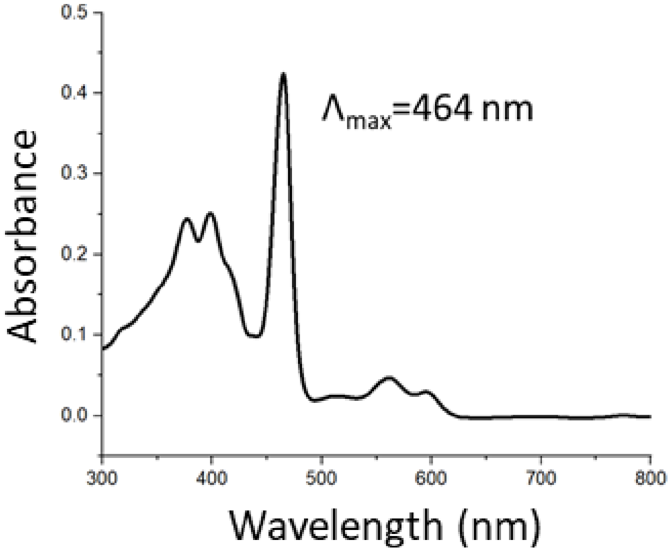

Figure s18. UV spectrum of $MnTPPS_4$ (λ_{max} = 464 nm, ε = 80,523 $M^{-1}cm^{-1}$) measured in PBS at 25 ºC.

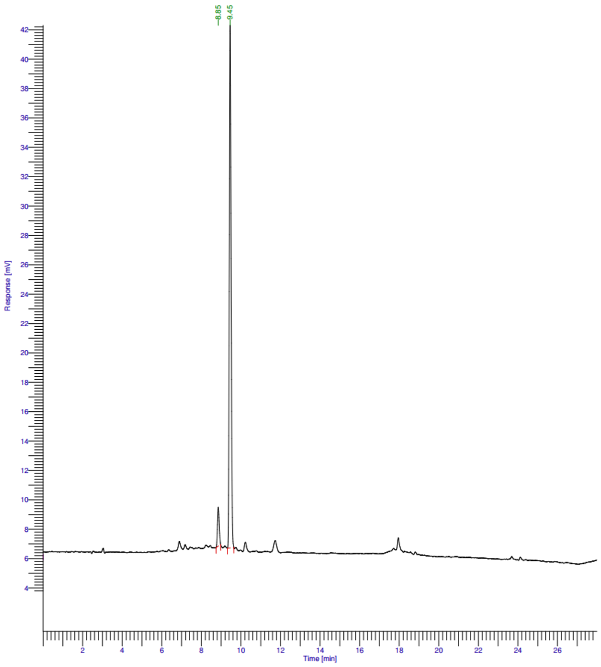

Figure s19. HPLC spectra of $TPPS_3NH_2$ were recorded using a **PerkinElmer Series 200 system** with UV/Vis detectors recording at 419 nm and using an acetonitrile and 10 mM ammonium acetate (NH_4OAc) gradient mix. Elution occurred at 9.45 minutes with 93.39% purity.

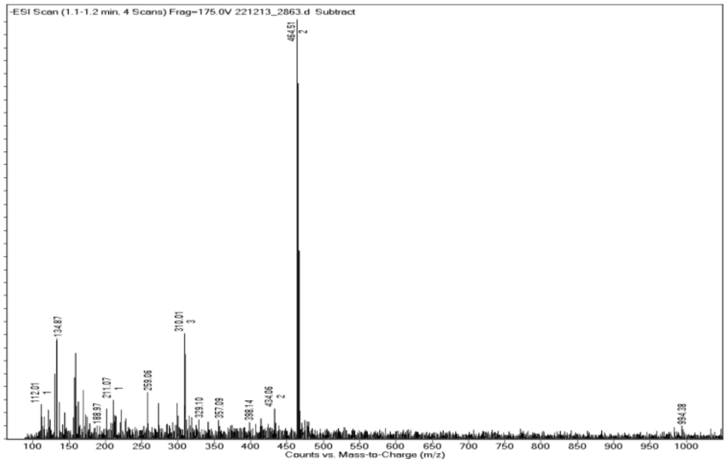

Target Ion Species

Ion Species	m/z	Ionic Formula
M-2	464.5135	C44 H27 N5 O9 S3 Zn

MFG Calculator Results

Target m/z	Ionic Formula	Calc m/z	+/- (mDa)	+/- (ppm)	MFG Score
464.5135	C43 H31 N O13 S3 Zn	464.5130	0.5	1.1	91.36
464.5135	C44 H27 N5 O9 S3 Zn	464.5137	-0.2	-0.4	90.97
464.5135	C31 H35 N3 O20 S3 Zn	464.5139	-0.4	-0.9	89.74
464.5135	C45 H23 N9 O5 S3 Zn	464.5143	-0.8	-1.7	88.61
464.5135	C32 H31 N7 O16 S3 Zn	464.5146	-1.1	-2.4	88.51
464.5135	C27 H31 N9 O18 S3 Zn	464.5126	0.9	1.9	87.43
464.5135	C39 H27 N7 O11 S3 Zn	464.5117	1.8	3.9	85.64
464.5135	C38 H31 N3 O15 S3 Zn	464.5110	2.5	5.4	80.79
464.5135	C49 H27 N3 O7 S3 Zn	464.5157	-2.2	-4.7	78.36

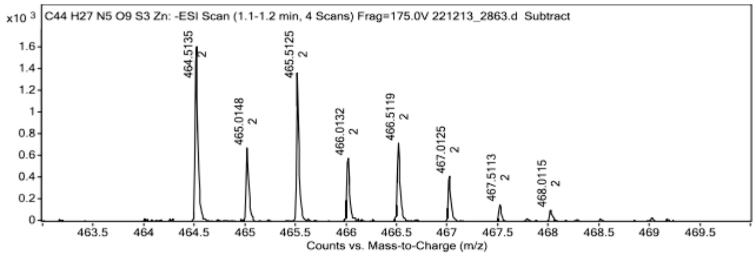

Predicted Isotope Match Table

Isotope	m/z	Calc m/z	Diff (mDa)	Abund (%)	Calc Abund (%)	+/-
1	464.5135	464.5137	-0.2	100.0	100.0	0.0
2	465.0148	465.0152	-0.4	44.0	52.4	8.4
3	465.5125	465.5128	-0.3	83.5	86.7	3.2
4	466.0132	466.0136	-0.4	36.4	49.1	12.7
5	466.5119	466.5121	-0.2	44.2	63.7	19.5
6	467.0125	467.0131	-0.6	25.9	29.8	3.9
7	467.5113	467.5122	-0.9	9.8	15.5	5.7
8	468.0115	468.0126	-1.1	6.6	5.5	-1.1

Figure s20. Mass Spectrum obtained for $ZnTPPS_3NH_2$ with an Agilent 6538 Q-TOF system in ESI MS Negative mode. MS (ESI) *m/z* calculated for $[M_2\text{-}6H]^{2-}$ $((C_{44}H_{27}N_5O_9S_3Zn)_2)^{2-}$: 464.5137. Found: 464.5135. Is consistent with dimers formed by sulfonatophenyl metalloporphyrins.

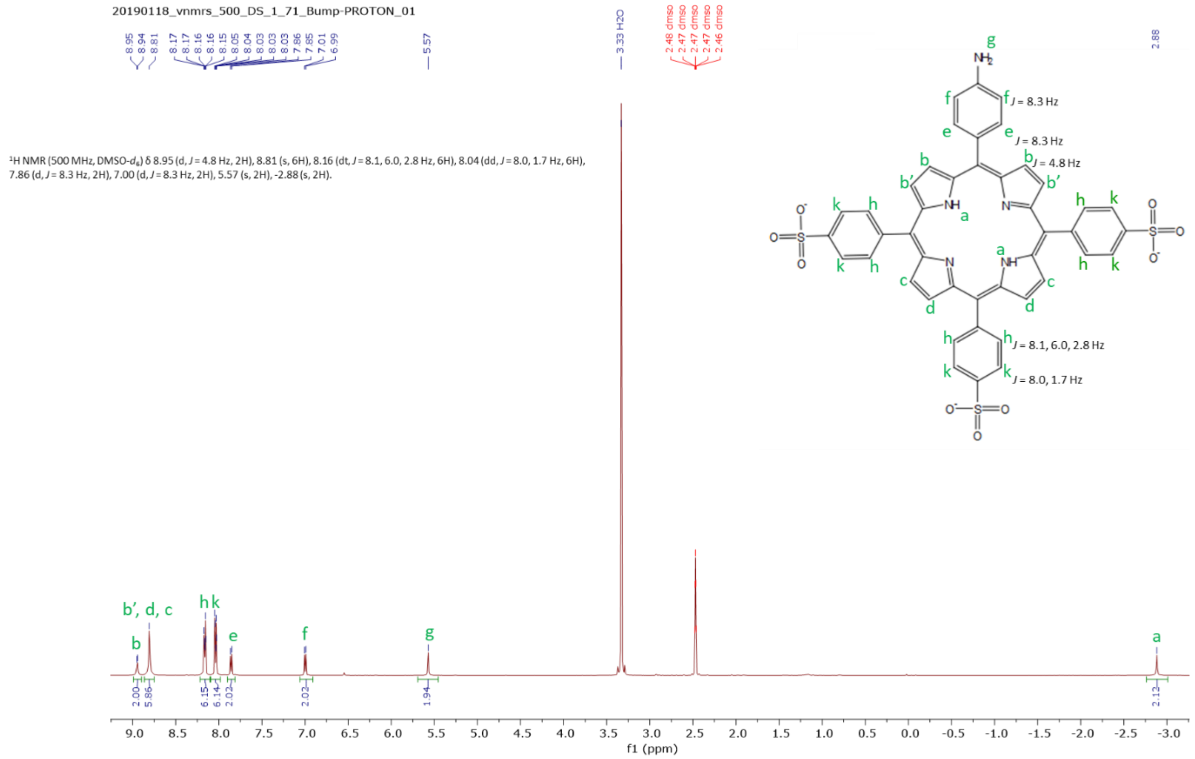

Figure s21. H[1]NMR spectrum of $TPPS_3NH_2$ acquired on Bruker US 500 MHz system. Chemical shifts and splitting pattern are consistent with literature. ^{1}H NMR (500 MHz, DMSO-d_6) δ 8.95 (d, J = 4.8 Hz, 2H), 8.81 (s, 6H), 8.16 (dt, J = 8.1, 6.0, 2.8 Hz, 6H), 8.04 (dd, J = 8.0, 1.7 Hz, 6H), 7.86 (d, J = 8.3 Hz, 2H), 7.00 (d, J = 8.3 Hz, 2H), 5.57 (s, 2H), -2.88 (s, 2H).

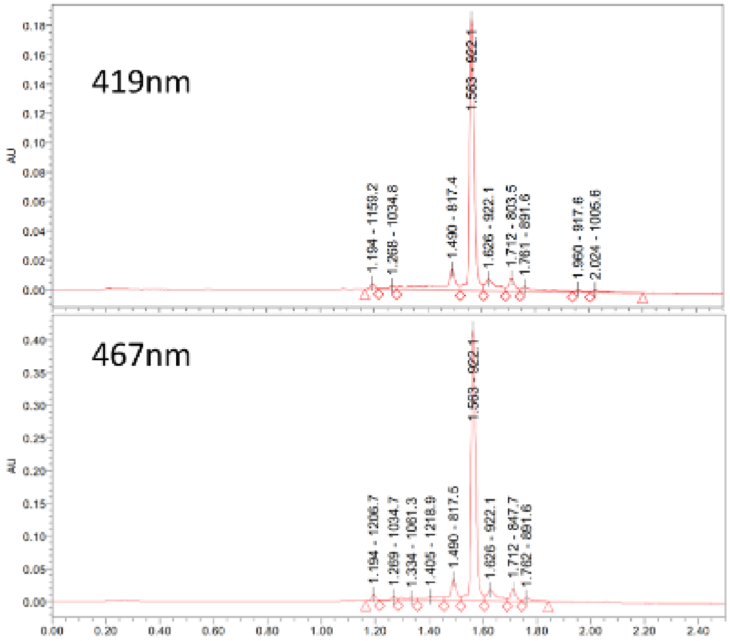

Figure s22. HPLC of $MnTPPS_3NH_2$ eluted at 1.563 min λ_{max}= 469 nm.

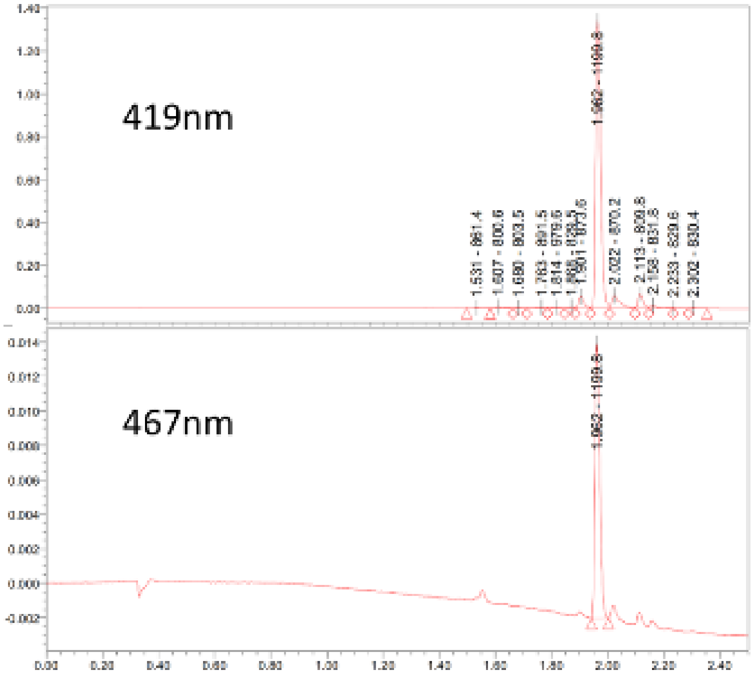

Figure s23. HPLC of $TPPS_3NH_2$ eluted at 1.962 min λ_{max}= 417 nm.

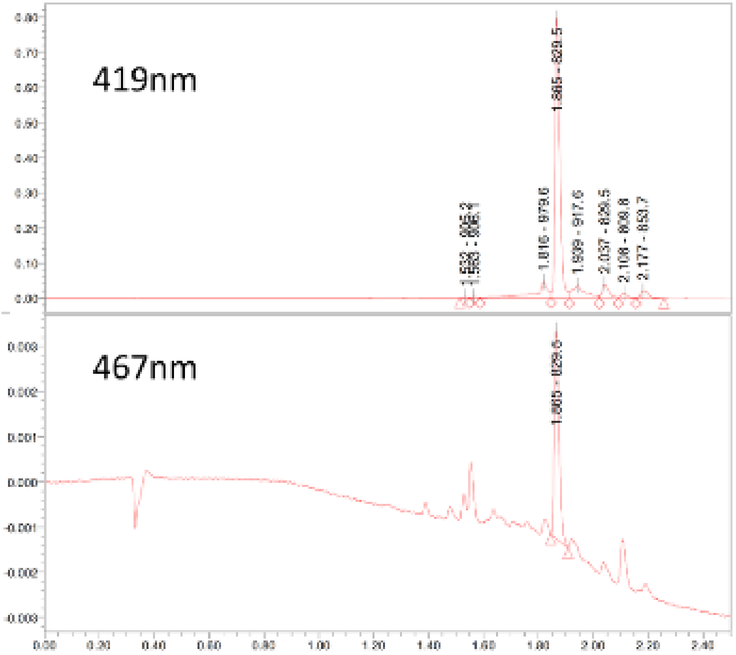

Figure s24. UHPLC of $ZnTPPS_3NH_2$ eluted at 1.865 min λ_{max}= 424 nm.

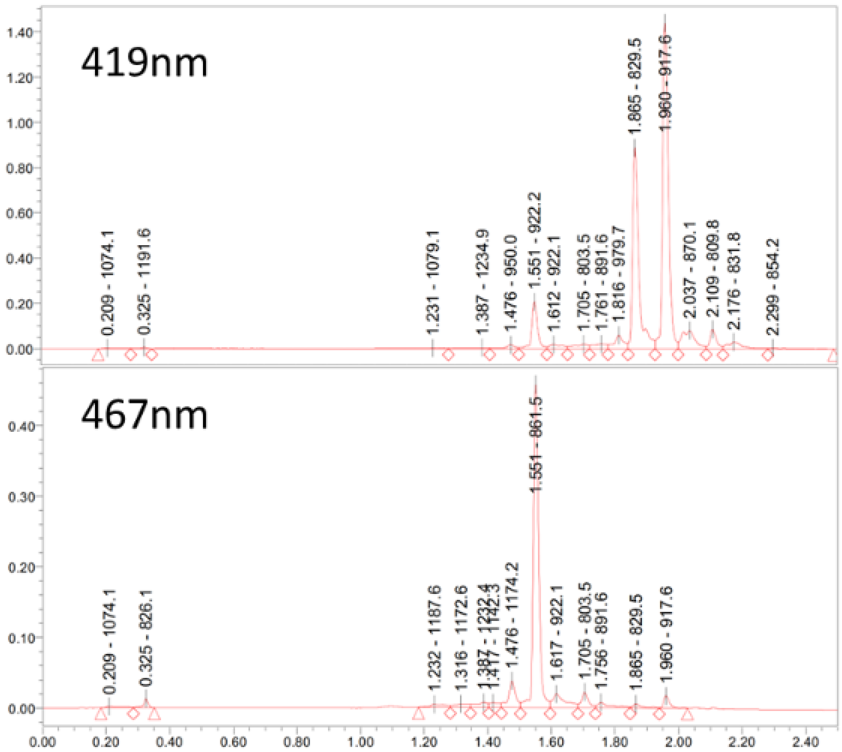

Figure s25. UHPLC of mixture of $MnTPPS_3NH_2$ eluted at 1.551 min λ_{max}= 469.1 nm, $ZnTPPS_3NH_2$ eluted at 1.865 min λ_{max}= 424.3 nm, and $TPPS_3NH_2$ eluted at 1.960 min λ_{max}= 417 nm.

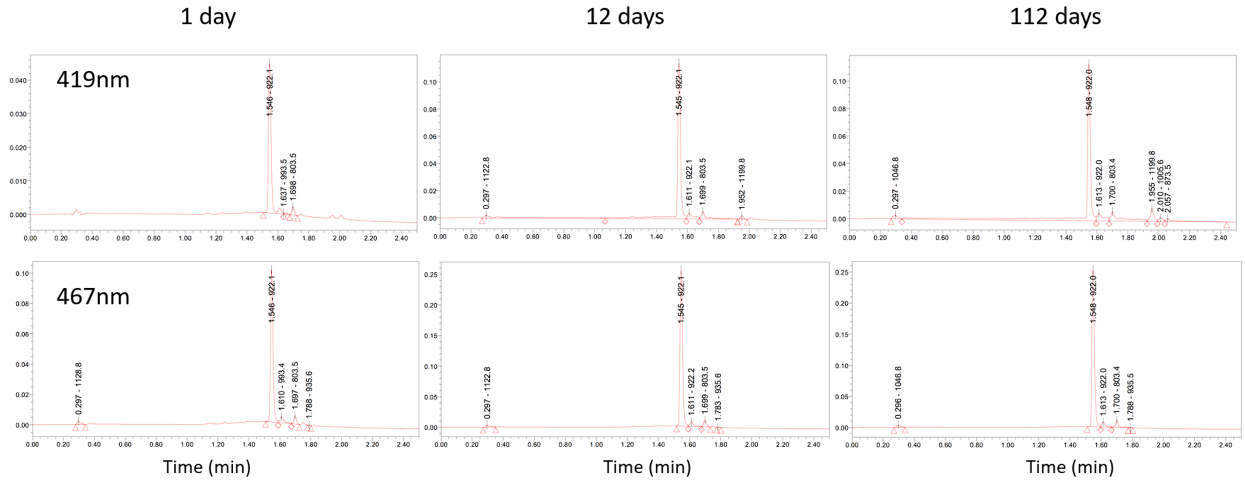

Figure s26. Example UHPLC spectrum of $MnTPPS_3NH2$ stress tested with 1M HCl over 112 days. $TPPS_3NH_2$ was detected at 12 and 112 days, eluting at 1.95 minutes λ_{max}= 417.1 nm, suggesting demetalation of 0.33 ± 0.1 and 1.64 ± 0.3 % after 12 and 112 days.

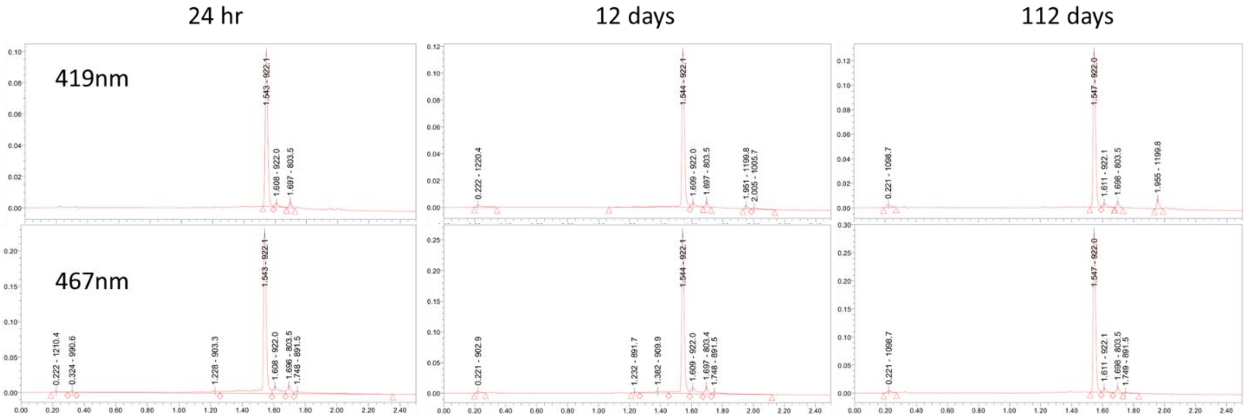

Figure s27. Example UHPLC spectrum of MnTPPS$_3$NH2 stress tested with 0.1M HCl over 112 days. TPPS$_3$NH$_2$ was detected at only 112 days, eluting at 1.96 minutes λ_{max}= 417.1 nm, suggesting demetalation of 0.93% after 112 days.

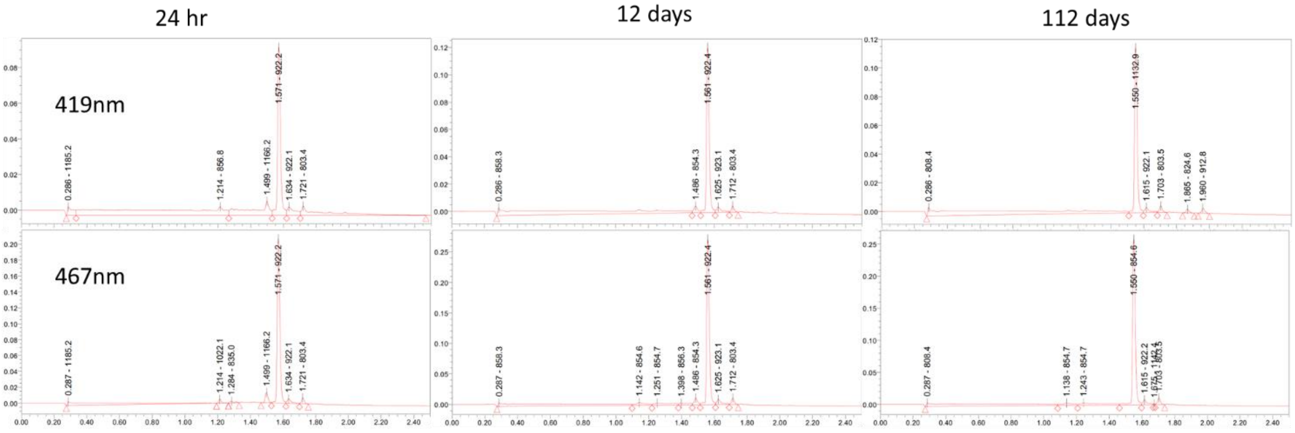

Figure s28. Example UHPLC spectrum of MnTPPS$_3$NH2 stress tested with 10 M ZnCl$_2$ over 112 days. ZnTPPS$_3$NH$_2$ was only detected after 112 days, eluting at 1.865 minutes λ_{max}= 424.3 nm with TPPS$_3$NH$_2$ also detected at 112 days, eluting at 1.96 minutes λ_{max}= 417.1 nm with these peaks associated with 0.33 ± 0.07% trans-metalation and 0.47 ± 0.07% demetalation.

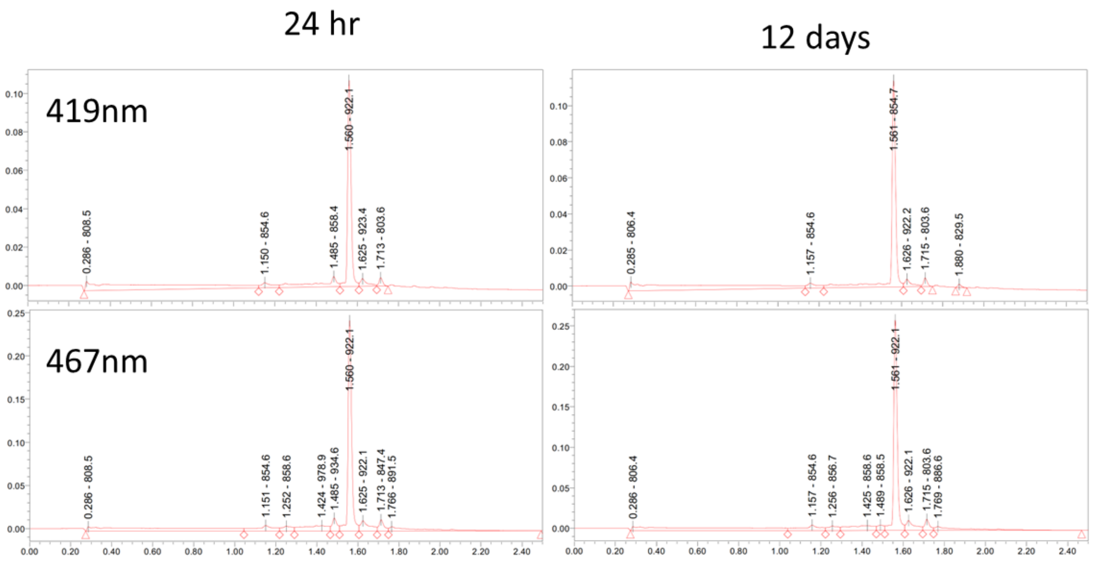

Figure s29. Example UHPLC spectrum of $MnTPPS_3NH_2$ stress tested with 10 M $ZnCl_2$ and 0.1M HCl over 12 days. $ZnTPPS_3NH_2$ was detected at 12 days, eluting at 1.88 minutes λ_{max}= 424.3 nm, suggesting demetalation of 0.2 ± 0.08% after 12 days.

Characterization of HSA

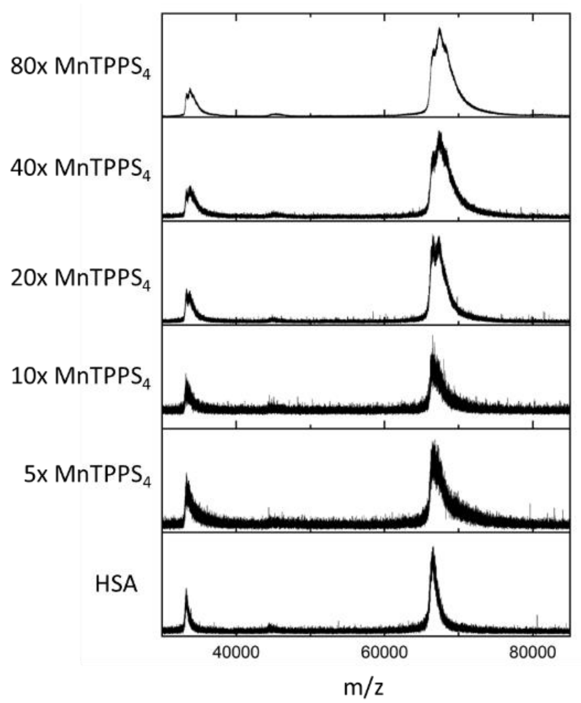

Figure s30. MALDI-TOF/MS of $MnTPPS_4$ labeled HSA. Average *m/z* for HSA was found to be 66,500 Da. Average *m/z* of product from reaction using 5x $MnTPPS_4$ was found to be 66,600 Da, suggesting 0.09 $MnTPPS_4$ per HSA and tagging efficiency of 1.8%. Average *m/z* of product from reaction using 10x $MnTPPS_4$ was found to be 66,600 Da, suggesting 0.09 $MnTPPS_4$ per HSA and tagging efficiency of 0.9%. Average *m/z* of product from reaction using 20x $MnTPPS_4$ was found to be 67,300 Da, suggesting 0.8 $MnTPPS_4$ per HSA and tagging efficiency of 4%. Average *m/z* of product from reaction using 40x $MnTPPS_4$ was found to be 67,400 Da, suggesting 0.8 $MnTPPS_4$ per HSA and tagging efficiency of 2%. Average *m/z* of product from reaction using 80x $MnTPPS_4$ was found to be 67,400 Da, suggesting 0.8 $MnTPPS_4$ per HSA and tagging efficiency of 1%.

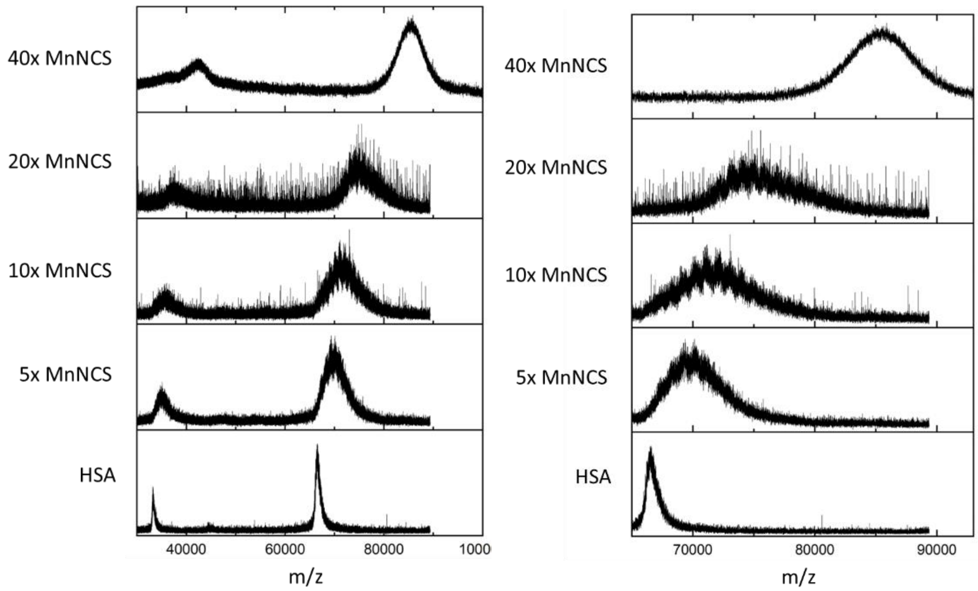

Figure s31. MALDI-TOF/MS of $MnTPPS_3NCS$ labeled HSA. Average *m/z* for HSA was found to be 66,500 Da. Average *m/z* of product from reaction using 5x $MnTPPS_3NCS$ was found to be 70,200 Da, suggesting 3.5 $MnTPPS_3$ per HSA and tagging efficiency of 70%. Average *m/z* of product from reaction using 10x $MnTPPS_3NCS$ was found to be 72,500 Da, suggesting 6 $MnTPPS_3$ per HSA and tagging efficiency of 60%. Average *m/z* of product from reaction using 20x $MnTPPS_3NCS$ was found to be 76,000 Da, suggesting 9.5 $MnTPPS_3$ per HSA and tagging efficiency of 48%. Average *m/z* of product from reaction using 40x $MnTPPS_3NCS$ was found to be 85,500 Da, suggesting 19 $MnTPPS_3$ per HSA and tagging efficiency of 48%.

Table s1. Previously published results of fluorophore conjugation to human serum albumin.

	Chemistry	Equiv. Added to HSA [Conjugate]/[HSA]	Avg. degree of labeling [Conjugate]/[HSA]	Tagging efficiency (%)	Ref
FITC	Isothiocyanate	21.4	3	14.0	44
RITC		6.2	2	32.3	
		12.4	3.2	25.8	
		21.3	4.9	23.0	
		24.8	6	24.2	
		62.0	3.2	5.2	

Table s2. Previously published results obtained for the conjugation of chelating agents to albumin.

	Chemistry	Reaction [Conjugate]/[HSA]	Product [Conjugate]/[HSA]	Tagging efficiency (%)	Metal	ref
**p-SCN-Bn-DOTA/BSA**	Isothiocyanate	40	4.1	10.3	Eu	45
**p-SCN-Bn-DOTA**		20	3.05 ± 0.23	15.2	N/A	46
**p-SCN-Bn-DOTA**		23	6.17 ± 0.38	20.8		
**DTPA-SCN**		5	2 (biased by removal of unbound)	Unknown, removed unbound albumin	Tb	47
**p-SCN-Bn-DOTA**						
**SCN-NETA**						
**SCN-DTPA**						
**p-SCN-C-DOTA**		110	16	14.5	Gd	35
**p-SCN-C-DOTA[Gd]**	Pre-metalated isothiocyanate	110	18	16.3	Gd	
**Dianhydride c-DTPAA**	Dianhydride	200	19	9.5	Gd	48
**DTPA**	N,N'-dicyclohexylcarbodiimide coupling	6800	25 (BSA)	0.37	Gd	49
		2x 6800x reactions	45	0.26		
**NH_2–Bn–DTPA**	bromoacetic acid, 2,3,5,6-tetrafluorophenyl ester activation of HSA	90.8	14	15.4	Gd	50
	glutarylated albumin	679.3	36	5.3	Gd	
	glutaraldehyde coupling	85.9	5	5.8	Gd	

General UHPLC procedure. UHPLC was performed on a Waters ACQUITY H-class UHPLC system with C18 BEH column with 1.7 um particle size and 2.1 x 50 mm dimensions and PDA detector and single quadrupole MS detector at the University of Toronto Department of Chemistry ANALEST facility. The column was equilibrated with 10 mM ammonium acetate and 10% acetonitrile for 1 minute before sample injection. Samples were eluted with a gradient to 100% acetonitrile over 3 minutes at 0.6 mL/min.

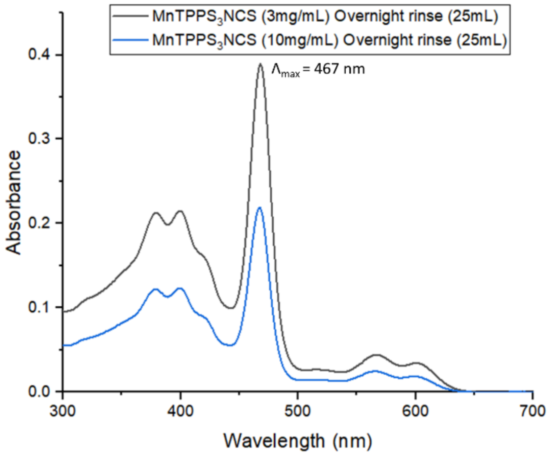

Figure s32. Example of UV-VIS spectrum of rinse solution of $MnTPPS_3NCS$-labeled collagen measured in PBS at 25 ^oC. Concentration is determined by measuring absorbance at 467 nm.

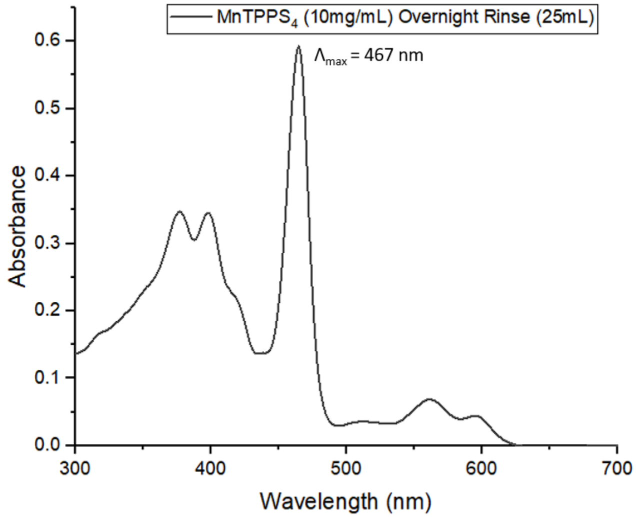

Figure s33. Example of UV-VIS spectrum of rinse solution of $MnTPPS_4$-labeled collagen measured in PBS at 25 ^oC. Concentration is determined by measuring absorbance at 467 nm.

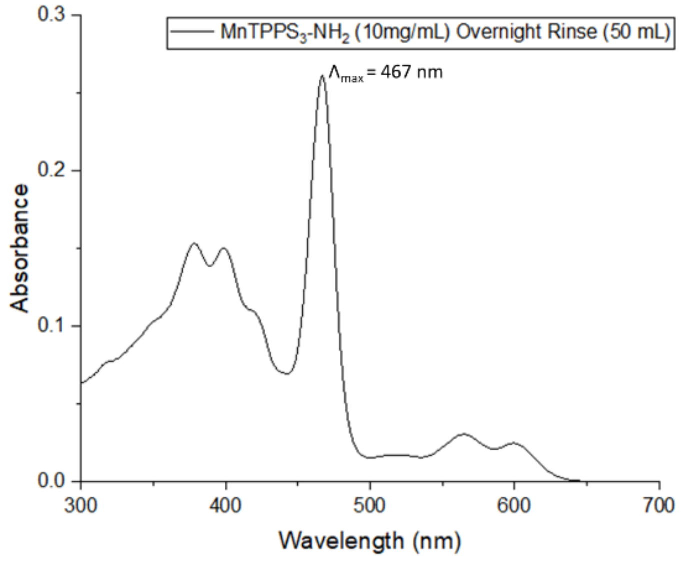

Figure s34. Example of UV-VIS spectrum of rinse solution of $MnTPPS_3NH_2$-labeled collagen measured in PBS at 25 °C. Concentration is determined by measuring absorbance at 467 nm.

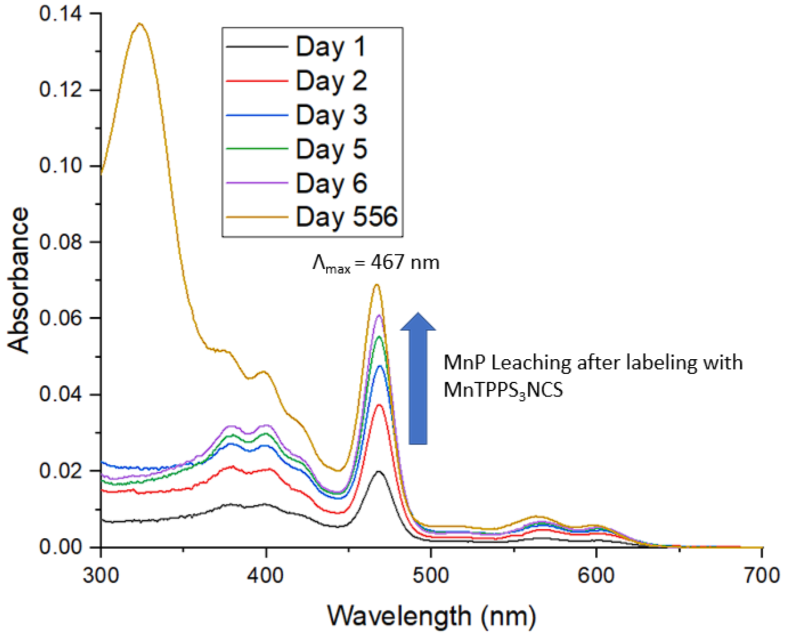

Figure s35. Example of UV-VIS spectrum monitoring porphyrin leaching from $MnTPPS_3NCS$-labeled collagen (10mg/mL) measured in PBS at 25 °C. Concentration was determined by measuring absorbance at 467 nm.

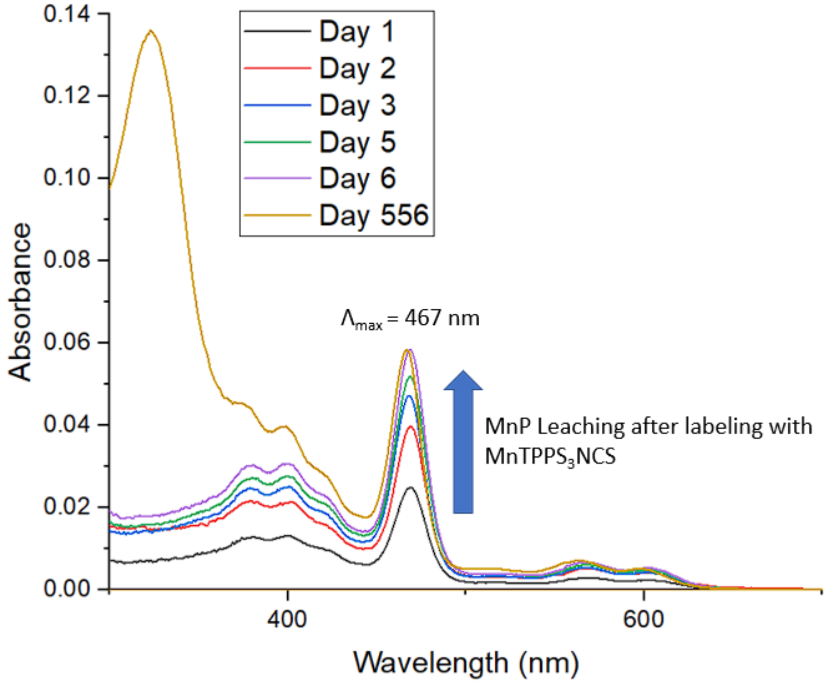

Figure s36. Example of UV-VIS spectrum monitoring porphyrin leaching from $MnTPPS_3NCS$-labeled collagen (3mg/mL) measured in PBS at 25 ºC. Concentration was determined by measuring absorbance at 467 nm.

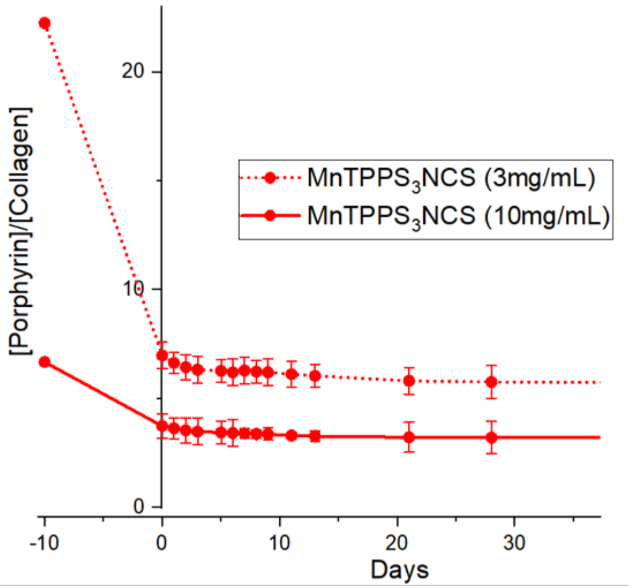

Figure s37. Moles of porphyrin per theoretical mole of collagen using 333 kD as molecular weight. Results calculated from % binding determined from UV-VIS and ICP-OES.

.

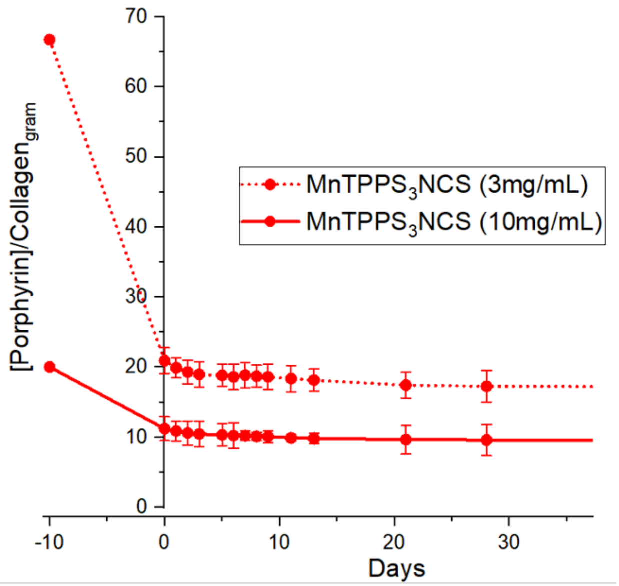

Figure s38. Moles of porphyrin per gram collagen. Results calculated from % binding determined from UV-VIS and ICP-OES.

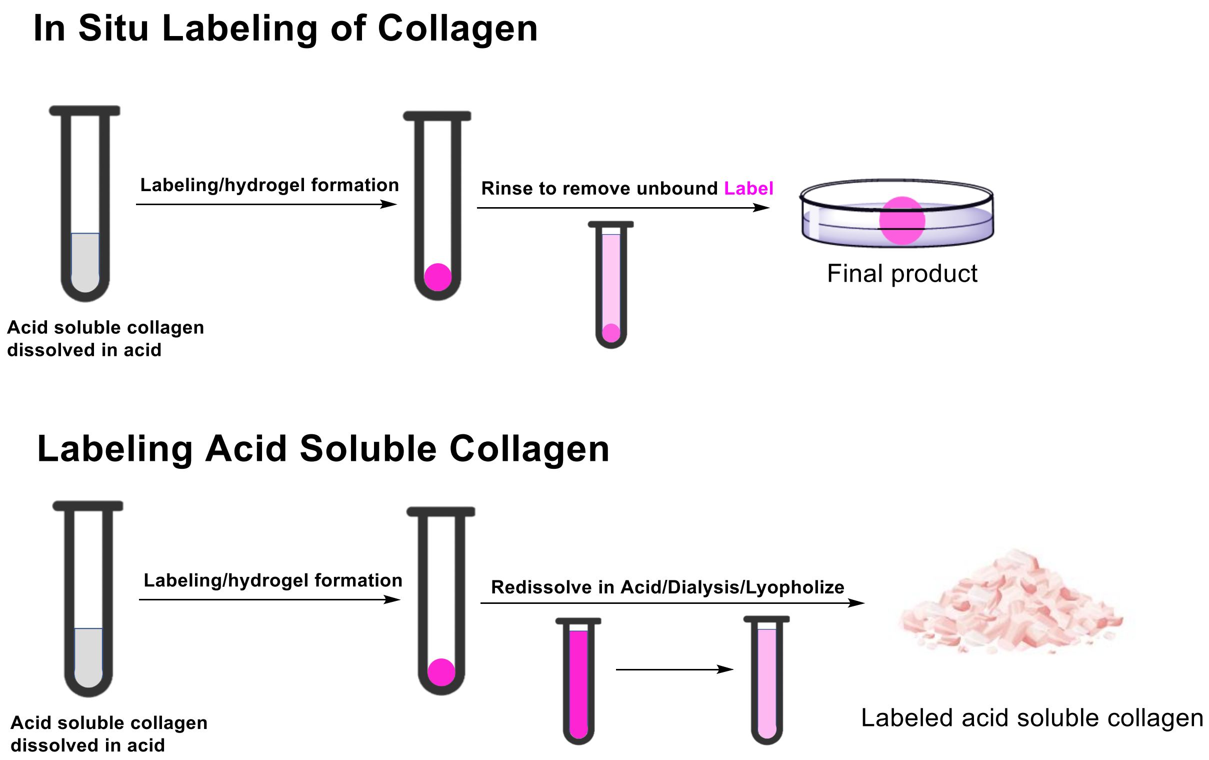

Figure s39. Difference between collagen labeling in situ and the synthesis of labeled acid-soluble collagen. The protocol for T_1 labeled acid-soluble collagen is as follows. All steps were performed at 4 °C. Lyophilized collagen was dissolved overnight in a solution of 0.1 % (w/v) acetic acid solution with vigorous stirring, forming a 3mg/mL collagen solution. Collagen was moved into a 50 mL falcon tube before $MnTPPS_3$-NCS porphyrin was added (at 3× stoichiometric ratio) and vortexed, followed by the slow dropwise addition of 0.5 M Na_2CO_3 with intermediate shaking until PH ≈ 9. This was left for 1 hour before reaction was stopped by adding a 3× volume of 4 °C PBS to falcon tubes and centrifuging at 16,000 rpm to pelletized collagen and unreacted porphyrin solution removed. This rinsing procedure was repeated three times before gels were transferred to a beaker and a 5× volume of 0.5 M acetic acid was added to dissolve collagen with stirring overnight. Salt and excess acetic acid was then removed by dialyzing collagen solution in 50 kDa Mw cut-off tubing against 50 mM acetic acid in deionized water. Dialysis was performed for a week with dialysate replaced at least 2 times a day with 50 mM solution of acetic acid. The resulting desalted collagen solution was then centrifuged at 16,000 rpm for 2 hours to remove any remaining insoluble collagen before supernatant was collected as a green solid, lyophilized, and stored at -80 °C. For the labeling of collagen gels, dried labelled collagen was dissolved to make 10mg/mL in 0.1 % (w/v) acetic acid overnight with stirring at 4 °C. This labelled collagen solution was blended with 10 mg/mL unlabelled collagen solution (advanced biomatrix) at 25 % (v/v). $MnTPPS_3NCS$ labeled gels were solidified by dispensing 0.5 mL into a falcon tube containing 2 mL of freshly prepared 0.2 M Na_2CO_3 on ice. Gels were then left in the fridge for 24 hours at 4 °C to solidify before being transferred to 50mL falcon tubes filled with PBS and left in an incubator at 37 °C for 24 hours before solution was replaced with fresh PBS.
